# Supplementary material for: Spatial distribution and determinants of unimproved sanitation facilities among households in Somalia: Using Somalia integrated household budget survey (SIHBS 2022)
Source: PLoS One. 2026 Apr 24;21(4):e0348029. doi: 10.1371/journal.pone.0348029 (PMC13108892; doi:10.1371/journal.pone.0348029)
Supplement: Attachment — Submitted filename: PONE-D-25-58376-AF.pdf [file pone.0348029.s002.pdf]

PLOS ONE

Spatial Distribution and Determinants of Unimproved Sanitation Facilities Among Households in Somalia: Using Somalia Integrated Household Budget Survey (SIHBS 2022)

--Manuscript Draft--

**Manuscript Number:**	PONE-D-25-58376
**Article Type:**	Research Article
**Full Title:**	Spatial Distribution and Determinants of Unimproved Sanitation Facilities Among Households in Somalia: Using Somalia Integrated Household Budget Survey (SIHBS 2022)
**Short Title:**	Spatial Distribution and Determinants of Unimproved Sanitation Facilities Among Households in Somalia
**Corresponding Author:**	Omar Muhumed Maidhane, M.D<br>Amoud University<br>Galkayo, Galmudug SOMALIA
**Keywords:**	Keywords: Unimproved sanitation; Spatial analysis; Multilevel modeling; Somalia; Public health disparities
**Abstract:**	Abstract<br>Background: Access to adequate sanitation is a critical public health issue and a fundamental human right, yet it remains a significant challenge in Somalia due to conflict, climate shocks, and political instability. A substantial portion of the population, particularly in rural and nomadic communities, relies on unimproved sanitation facilities, leading to a high burden of waterborne diseases and other health risks. This study aimed to assess the spatial distribution of unimproved sanitation and identify its individual and community-level determinants using recent national data.<br>Methods: This study conducted a secondary analysis of data from the 2022 Somalia Integrated Household Budget Survey (SIHBS), a nationally representative survey that included 7,212 households. The primary outcome was the use of unimproved sanitation facilities. A multilevel logistic regression model was employed to analyze the association between individual and community-level factors and unimproved sanitation. Geospatial analysis, including Global Moran's I for spatial autocorrelation and Getis-Ord Gi* for hotspot analysis, was used to identify the spatial patterns of unimproved sanitation.<br>Result: Overall, 36.87% of Somali households use unimproved sanitation facilities. There are significant disparities across residence types, with the highest prevalence among nomadic populations (83.28%), followed by rural (51.10%) and urban (23.88%) residents. The multilevel analysis revealed that households in permanent/formal housing (AOR: 3.42) and those with IDP status (AOR: 3.18) had significantly higher odds of using unimproved sanitation. At the community level, urban residence was paradoxically associated with higher odds of unimproved sanitation (AOR: 7.99) compared to rural areas, while nomadic populations had significantly lower odds (AOR: 0.04), likely reflecting a high prevalence of open defecation not captured as a "facility." Spatial analysis identified significant hotspots of unimproved sanitation in the Hiraan (90.65%) and Bay (80.39%) regions, and coldspots in Banadir (5.37%) and Lower Shabelle (3.70%).<br>Conclusion: The findings highlight deep inequalities in sanitation access across Somalia, driven by geographic location, socioeconomic status, and population group. The high prevalence of unimproved sanitation, especially among nomadic, rural, and displaced populations, calls for urgent, geographically-targeted interventions. A multi-pronged approach is necessary, focusing on the specific needs of different communities and addressing the underlying structural and individual-level drivers of poor sanitation to advance public health and sustainable development goals in the region.
**Order of Authors:**	Omar Muhumed Maidhane, M.D
	Omran Salih, PhD
	Abdisalan Hassan Muse, PhD

Powered by Editorial Manager® and ProduXion Manager® from Aries Systems Corporation

	Abdirahman Omer Osman
	Muse H Abdi, M.D
	Mahdi Hashi Hassan
	Nur Mohamud Ali
	Shacban Abdillahi Elmi
**Opposed Reviewers:**	
**Additional Information:**	
**Question**	**Response**
**Financial Disclosure**<br><br>Enter a financial disclosure statement that describes the sources of funding for the work included in this submission. Review the submission guidelines for detailed requirements. View published research articles from *PLOS ONE* for specific examples.<br><br>This statement is required for submission and **will appear in the published article** if the submission is accepted. Please make sure it is accurate.<br><br>**Funded studies**<br>Enter a statement with the following details:<br>• Initials of the authors who received each award<br>• Grant numbers awarded to each author<br>• The full name of each funder<br>• URL of each funder website<br>• Did the sponsors or funders play any role in the study design, data collection and analysis, decision to publish, or preparation of the manuscript?<br><br>Did you receive funding for this work?	The author(s) received no specific funding for this work.
**Competing Interests**<br><br>Use the instructions below to enter a competing interest statement for this submission. On behalf of all authors, disclose any competing interests that could be perceived to bias this work—acknowledging all financial support and any other relevant financial or non-financial competing interests.	The authors have declared that no competing interests

This statement is required for submission and **will appear in the published article** if the submission is accepted. Please make sure it is accurate and that any funding sources listed in your Funding Information later in the submission form are also declared in your Financial Disclosure statement.<br><br>View published research articles from *PLOS ONE* for specific examples.<br><br>**NO authors have competing interests**<br><br>Enter: *The authors have declared that no competing interests exist.*<br><br>**Authors with competing interests**<br><br>Enter competing interest details beginning with this statement:<br><br>*I have read the journal's policy and the authors of this manuscript have the following competing interests: [insert competing interests here]*<br><br>* typeset	
**Ethics Statement**<br><br>Enter an ethics statement for this submission. This statement is required if the study involved:<br><br>• Human participants<br>• Human specimens or tissue<br>• Vertebrate animals or cephalopods<br>• Vertebrate embryos or tissues<br>• Field research<br><br>Write "N/A" if the submission does not require an ethics statement.<br><br>General guidance is provided below. Consult the submission guidelines for detailed instructions. **Make sure that all**	No

Powered by Editorial Manager® and ProduXion Manager® from Aries Systems Corporation

**information entered here is included in the Methods section of the manuscript.**<br><br>**Format for specific study types**<br><br>**Human Subject Research (involving human participants and/or tissue)**<br>• Give the name of the institutional review board or ethics committee that approved the study<br>• Include the approval number and/or a statement indicating approval of this research<br>• Indicate the form of consent obtained (written/oral) or the reason that consent was not obtained (e.g. the data were analyzed anonymously)<br><br>**Animal Research (involving vertebrate animals, embryos or tissues)**<br>• Provide the name of the Institutional Animal Care and Use Committee (IACUC) or other relevant ethics board that reviewed the study protocol, and indicate whether they approved this research or granted a formal waiver of ethical approval<br>• Include an approval number if one was obtained<br>• If the study involved *non-human primates*, add *additional details* about animal welfare and steps taken to ameliorate suffering<br>• If anesthesia, euthanasia, or any kind of animal sacrifice is part of the study, include briefly which substances and/or methods were applied<br><br>**Field Research**<br><br>Include the following details if this study involves the collection of plant, animal, or other materials from a natural setting:<br>• Field permit number<br>• Name of the institution or relevant body that granted permission	
**Data Availability**<br><br>Authors are required to make all data underlying the findings described fully available, without restriction, and from the time of publication. PLOS allows rare exceptions to address legal and ethical	No - some restrictions will apply

concerns. See the PLOS Data Policy and FAQ for detailed information.<br><br>A Data Availability Statement describing where the data can be found is required at submission. Your answers to this question constitute the Data Availability Statement and **will be published in the article**, if accepted.<br><br>**Important:** Stating 'data available on request from the author' is not sufficient. If your data are only available upon request, select 'No' for the first question and explain your exceptional situation in the text box.<br><br>Do the authors confirm that all data underlying the findings described in their manuscript are fully available without restriction?	
**Describe where the data may be found in full sentences. If you are copying our sample text, replace any instances of XXX with the appropriate details.**<br><br>• If the data are **held or will be held in a public repository**, include URLs, accession numbers or DOIs. If this information will only be available after acceptance, indicate this by ticking the box below. For example: *All XXX files are available from the XXX database (accession number(s) XXX, XXX.*).<br>• If the data are all contained **within the manuscript and/or Supporting Information files**, enter the following: *All relevant data are within the manuscript and its Supporting Information files.*<br>• If neither of these applies but you are able to provide **details of access elsewhere**, with or without limitations, please do so. For example:<br><br>*Data cannot be shared publicly because of [XXX]. Data are available from the XXX Institutional Data Access / Ethics Committee (contact via XXX) for researchers who meet the criteria for access to confidential data.*	The data underlying the results presented in the study are available from the Somali National Bureau of Statistics (SNBS).[1][2][3] The dataset, the Somalia Integrated Household Budget Survey (SIHBS) 2022, can be requested by researchers through the Somali National Data Archive (SoNADA) portal on the SNBS website.[4]

Powered by Editorial Manager® and ProduXion Manager® from Aries Systems Corporation

The data underlying the results presented in the study are available from (include the name of the third party and contact information or URL).<br>• This text is appropriate if the data are owned by a third party and authors do not have permission to share the data.<br><br>* typeset	
Additional data availability information:	

Powered by Editorial Manager® and ProduXion Manager® from Aries Systems Corporation

Cover Letter

Omran Salih
Amoud University
omran.salih@amoud.edu.so

Date: 27/10/2025

To: Editor-in-Chief
PLOS One

Dear Editor,

I am pleased to submit our original research manuscript entitled *“Spatial Distribution and Determinants of Unimproved Sanitation Facilities Among Households in Somalia: Using Somalia Integrated Household Budget Survey (SIHBS 2022)”* for consideration for publication in PLOS One Journal.

This study addresses a pressing public health concern in Somalia, inadequate sanitation by employing both multilevel and geospatial analytical approaches. Using nationally representative data from the SIHBS 2022, we examined the spatial distribution and determinants of unimproved sanitation facilities across Somali households. The analysis identified substantial geographic disparities and highlighted vulnerable population groups, including nomadic and internally displaced communities, who are disproportionately affected.

Our findings contribute to the limited body of evidence on sanitation inequalities in fragile and conflict-affected settings. The study provides actionable insights for policymakers and development partners to design geographically targeted and context-specific interventions aimed at achieving Sustainable Development Goal 6 (Clean Water and Sanitation).

We believe this manuscript aligns well with the journal’s scope and audience, as it provides novel evidence linking spatial, social, and environmental determinants of health outcomes in low-resource and conflict-affected contexts. The manuscript is original, has not been published elsewhere, and is not under consideration by any other journal.

We sincerely hope that you find this study valuable and suitable for publication in PLOS One journal. Thank you for considering our submission. We look forward to your positive response.

Sincerely,
Omar

On behalf of all co-authors

Manuscript
Click here to access/download;Manuscript;Sanitation_OS_V0.docx

Spatial Distribution and Determinants of Unimproved Sanitation Facilities Among Households in Somalia: Using Somalia Integrated Household Budget Survey (SIHBS 2022)

Omar Muhumed Maidhane[1,3], Omran Salih[1,4,*], Abdisalam Hassan Muse[1,2], Abdirahman Omer Osman[1], Muse H Abdi[1], Mahdi Hashi Hassan[1], Nur Mohamud Ali[1], Shacban Abdilahi Elmi[1]

[1]School of Postgraduate Studies and Research, Amoud University, Borama 25263, Somalia

[2]Research and Innovation Center, Amoud University, Borama 25263, Somalia

[3]Research and Innovation Center, Galkayo University, Galmudug, Somalia.

[4]Institute of System Science, Durban University of Technology, Durban, South Africa

[*]Corresponding Author: *Omar Muhumed Maidhane*

Abstract

Background: Access to adequate sanitation is a critical public health issue and a fundamental human right, yet it remains a significant challenge in Somalia due to conflict, climate shocks, and political instability. A substantial portion of the population, particularly in rural and nomadic communities, relies on unimproved sanitation facilities, leading to a high burden of waterborne diseases and other health risks. This study aimed to assess the spatial distribution of unimproved sanitation and identify its individual and community-level determinants using recent national data.

Methods: This study conducted a secondary analysis of data from the 2022 Somalia Integrated Household Budget Survey (SIHBS), a nationally representative survey that included 7,212 households. The primary outcome was the use of unimproved sanitation facilities. A multilevel logistic regression model was employed to analyze the association between individual and community-level factors and unimproved sanitation. Geospatial analysis, including Global Moran's I for spatial autocorrelation and Getis-Ord Gi* for hotspot analysis, was used to identify the spatial patterns of unimproved sanitation.

Result: Overall, 36.87% of Somali households use unimproved sanitation facilities. There are significant disparities across residence types, with the highest prevalence among nomadic populations (83.28%), followed by rural (51.10%) and urban (23.88%) residents. The multilevel analysis revealed that households in permanent/formal housing (AOR: 3.42) and those with IDP status (AOR: 3.18) had significantly higher odds of using unimproved sanitation. At the community level, urban residence was paradoxically associated with higher odds of unimproved sanitation (AOR: 7.99) compared to rural areas, while nomadic populations had significantly lower odds (AOR: 0.04), likely reflecting a high prevalence of open defecation not captured

as a "facility." Spatial analysis identified significant hotspots of unimproved sanitation in the Hiraan (90.65%) and Bay (80.39%) regions, and coldspots in Banadir (5.37%) and Lower Shabelle (3.70%).

Conclusion: The findings highlight deep inequalities in sanitation access across Somalia, driven by geographic location, socioeconomic status, and population group. The high prevalence of unimproved sanitation, especially among nomadic, rural, and displaced populations, calls for urgent, geographically-targeted interventions. A multi-pronged approach is necessary, focusing on the specific needs of different communities and addressing the underlying structural and individual-level drivers of poor sanitation to advance public health and sustainable development goals in the region.

Keywords: Unimproved sanitation; Spatial analysis; Multilevel modeling; Somalia; Public health disparities

1. Introduction

Access to adequate and equitable sanitation is a fundamental human right and a critical component of public health and sustainable development (Yus[illegible] et al., 2023a). Globally, 3.5 billion people still lacked safely managed sanitation in 2022, with 419 million practicing open defecation (Saleem et al., 2019). The disease burden resulting from inadequate sanitation is immense, contributing to the transmission of diseases like cholera, typhoid, and intestinal worm infections, which disproportionately affect children under five (Demoze et al., 2025; Unicef, 2018). Poor sanitation is a significant factor in environmental degradation and is closely linked to childhood stunting and malnutrition (Demoze et al., 2025; Pullan et al., 2014). The Sustainable Development Goals (SDGs), particularly Target 6.2, aim to achieve universal access to sanitation for all and end open defecation by 2030, paying special attention to the needs of women, girls, and those in vulnerable situations (Yusuf et al., 2023b).

Sub-Saharan Africa faces the most significant challenges in sanitation coverage, with disparities between urban and rural areas and slow overall progress (J. Brown et al., 2013; Spears et al., 2013). Somalia, in particular, confronts acute sanitation challenges exacerbated by decades of conflict, political instability, climate-related disasters such as recurrent droughts, and large-scale displacement (Ahmed & Ali, 2024; Yusuf et al., 2023b). According to the WHO/UNICEF Joint Monitoring Programme (JMP), access to even basic sanitation facilities in Somalia is critically low, with a significant portion of the population, especially in rural and nomadic communities, resorting to unimproved facilities or open defecation (ESCAP, 2016; Hopewell & Graham, 2014). Recent data from the 2020 Somalia ~~Health and Demographic~~ Survey indicated that a substantial proportion (59.7%) of households utilized unimproved sanitation facilities (Azanaw et al., 2023). This situation poses severe risks to public health, dignity, and safety, particularly for women and

girls who face heightened risks of harassment and violence when seeking private places to relieve themselves (Osumanu et al., 2019).

Despite the clear urgency, there is limited comprehensive research on the specific factors driving the prevalence of unimproved sanitation in Somalia. While several studies have highlighted the scale of the problem, a detailed analysis that combines both spatial and multilevel approaches to understand the geographic disparities and the interplay of individual and community-level determinants is lacking (Belay et al., 2022; Unicef, 2018). Such an analysis is crucial for designing evidence-based, targeted interventions that can address the root causes of poor sanitation. Therefore, this study aims to assess the spatial distribution and identify the determinants of unimproved sanitation facilities among households in Somalia, using recent data from the Somalia Integrated Household Budget Survey (SIHBS 2022).

This study provides a novel and in-depth analysis of sanitation challenges in Somalia by being one of the first to combine a multilevel modeling approach with advanced spatial analysis. While previous research has acknowledged the poor state of sanitation in the country, this study moves beyond descriptive statistics to simultaneously investigate both individual and community-level determinants of unimproved sanitation. The use of the recent and comprehensive 2022 Somalia Integrated Household Budget Survey (SIHBS) dataset allows for a more current and granular understanding of the issue. Furthermore, the application of geospatial techniques such as Getis-Ord Gi* hotspot analysis and spatial autocorrelation provides a clear visual and statistical identification of geographic disparities, pinpointing specific regions and communities that are most affected. This dual approach offers a more robust evidence base for policymakers and public health stakeholders, enabling the design of targeted, context-specific interventions that address both the socioeconomic drivers and the geographical clustering of poor sanitation in Somalia.

2. Material And Methods

2.1 Study Area

The study was conducted in Somalia; a country located in the Horn of Africa. It is bordered by Ethiopia to the west, Djibouti to the northwest, the Gulf of Aden to the north, the Indian Ocean to the east, and Kenya to the southwest. The country is organized as a federation of member states. Decades of civil conflict and environmental shocks have severely impacted its infrastructure, including water and sanitation systems, leading to widespread displacement and vulnerability among its population. The population is diverse, comprising urban, rural, and nomadic communities, each facing unique challenges in accessing basic services (Yusuf et al., 2023b).

2.2 Study Design and Setting

This study employed a secondary analysis of data derived from a community-based cross-sectional survey. Specifically, it utilized household-level data from the Somalia Integrated Household Budget Survey (SIHBS) 2022. The SIHBS was designed to be nationally representative, encompassing urban, rural, and nomadic populations across all federal member states of Somalia.

2.3 Data Source

The data for this study was sourced from the 2022 Somalia Integrated Household Budget Survey (SIHBS), conducted by the Somalia National Bureau of Statistics (SNBS) (Demsash et al., 2023).The SIHBS is the first comprehensive household budget survey conducted in the country since 1985. The survey employed a two-stage stratified cluster sampling design to ensure a representative sample. In the first stage, enumeration areas (EAs) were selected with probability proportional to their size. In the second stage, a fixed number of households were systematically selected from each EA. The SIHBS 2022 included a nationally representative sample of 7,212 households from urban, rural, and nomadic areas (Unicef, 2018). After data cleaning and management, this analysis included a final weighted sample representative of households across Somalia.

2.4 Data validation

To ensure the findings are representative of the national population and to account for the complex survey design, sampling weights provided in the SIHBS dataset were applied during all statistical analyses. This weighting procedure adjusts for the unequal probability of selection and non-response rates, ensuring reliable and valid estimates.

2.5 Study variables

The primary outcome variable was the status of the household sanitation facility, categorized as a binary variable: "unimproved" or "improved." This classification was based on the WHO/UNICEF JMP definitions (Organization, 2022). "Unimproved sanitation facilities" were defined as pit latrines without a slab or platform, hanging latrines, bucket latrines, and the practice of open defecation(C. Brown et al., 2016). *This was coded as '1'.* "Improved sanitation facilities" included flush or pour-flush toilets connected to a piped sewer system, septic tanks, or pit latrines; ventilated improved pit latrines; and pit latrines with a slab, which are not shared with other households. *This was coded as '0'.*

The independent variables were classified into individual-level and community-level factors. Individual-level variables included the sex of the household head (male or female), age of the household head (categorized), educational attainment (no education, primary, secondary, or higher), household size, and the household wealth index, which was categorized into five quintiles: poorest, poorer, middle, richer, and richest. Community-level variables comprised the place of residence (urban, rural, or nomadic), region or federal member state, and community media exposure, defined as the proportion of households within the enumeration area (EA) that had access to radio or television.

2.6 Data Analysis

2.6.1 Univariant and Multilevel Analysis

Data cleaning, coding, and analysis were performed using STATA version 17. Descriptive statistics, including frequencies and percentages, were used to summarize the characteristics of the study population and the prevalence of unimproved sanitation. Due to the hierarchical nature of the survey data, where households (level 1) are nested within enumeration areas or clusters (level 2), a multilevel logistic regression model was employed to identify the determinants of unimproved sanitation (Desye et al., 2025). This approach accounts for the clustering effect and avoids the underestimation of standard errors that can occur with standard logistic regression (Demoze et al., 2025; Heijnen et al., 2014).

Four consecutive models were fitted: a null model (Model 0) with no explanatory variables to assess the variance in unimproved sanitation between clusters; Model I included only individual-level variables; Model II included only community-level variables; and Model III, the final model, included both individual and community-level variables simultaneously. Measures of variation (random effects) such as the Intraclass Correlation Coefficient (ICC), Median Odds Ratio (MOR), and Proportional Change in Variance (PCV) were calculated to quantify the extent of clustering. Model fit was assessed using the Akaike information criterion (AIC) and Bayesian Information criterion (BIC), with the model having the lowest AIC and BIC values considered the best fit. Adjusted Odds Ratios (AOR) with 95% confidence intervals (CI) were used to report the final associations, and a p-value of < 0.05 was considered statistically significant.

2.6.2 Spatial Analysis

Geospatial analysis was conducted to explore the spatial distribution and identify geographic hotspots of unimproved sanitation facilities in Somalia using ArcGIS version 10.7 and SaTScan

version 10.1. First, spatial autocorrelation was assessed using the Global Moran's I statistic to determine if the pattern of unimproved sanitation was clustered, dispersed, or random. A statistically significant and positive Moran's I value indicates spatial clustering of similar values(Novotný & Mamo, 2022).

Second, Getis-Ord Gi* hotspot analysis was performed to identify the locations of statistically significant hot spots (areas with high prevalence of unimproved sanitation) and cold spots (areas with low prevalence) (Novotný & Mamo, 2022). Third, ordinary Kriging spatial interpolation was used to predict the prevalence of unimproved sanitation in unsampled areas based on the values from sampled clusters, creating a continuous risk map for the entire country. Finally, a purely spatial scan statistic using a Bernoulli model was employed in SaTScan software to detect significant spatial clusters of unimproved sanitation. Households with unimproved facilities were treated as cases, and those with improved facilities as controls, to identify geographic areas with a higher-than-expected prevalence.

2.7 Ethical Statement

This study is based on a secondary analysis of publicly available and anonymized data from the Somalia Integrated Household Budget Survey (SIHBS 2022). The original survey protocol was reviewed and approved by the relevant institutional review board in Somalia. The implementing agency, the Somalia National Bureau of Statistics (SNBS), obtained informed consent from all participants before data collection. Permission to use the dataset for this study was obtained through the official data archive channels.

3. Results

3.1 Univariate Analysis

The data illustrated in Table 1 presents the comprehensive analysis of sanitation facilities in Somalia reveals a complex interplay of individual and community-level factors, all demonstrating statistically significant associations with access to unimproved versus improved facilities, as evidenced by both percentage distributions and the magnitude of the chi-squared statistic and its corresponding p-value.

Overall, 36.87% of facilities are unimproved, while 63.13% are improved. At the individual level, the age of the household head shows a significant relationship, with a chi-squared value of 47.37 and a p-value less than 0.001, with unimproved sanitation peaking among those aged 50-59 (41.59%) and lowest in the youngest group (<20 years, 32.74%). Gender of the household head

is also a significant factor, with a chi-squared value of 12.78 and a p-value less than 0.001, as male-headed households exhibit a higher proportion of unimproved sanitation (38.58%) compared to female-headed households (35.42%). Education emerges as a powerful determinant, with a chi-squared value of 247.60 and a p-value less than 0.001, as households where the head attended school have considerably lower unimproved rates (30.12%) than those who did not (44.02%). Marital status is also highly significant, with a chi-squared value of 153.52 and a p-value less than 0.001, with married individuals experiencing the highest unimproved rates (42.66%). Housing type, with a chi-squared value of 1300 and a p-value less than 0.001, and lighting source, with a chi-squared value of 1200 and a p-value less than 0.001, are critical indicators, with temporary housing (61.32% unimproved) and traditional lighting (58.86% unimproved) demonstrating a strong association with poor sanitation.

Furthermore, ID[illegible]tatus, with a chi-squared value of 173.57 and a p-value less than 0.001, starkly highlights vulnerability, with 64.27% of IDP households having unimproved facilities. Access to modern amenities like the internet, with a chi-squared value of 691.78 and a p-value less than 0.001, and electricity, with a chi-squared value of 1600 and a p-value less than 0.001, significantly correlates with improved sanitation, as does food security, with a chi-squared value of 593.91 and a p-value less than 0.001, where food-insecure households report 46.76% unimproved facilities. While statistically significant, with a chi-squared value of 3.66 and a p-value of 0.056, the impact of labor status on sanitation is comparatively smaller.

At the community level, residence type profoundly influences sanitation access, with a chi-squared value of 1800 and a p-value less than 0.001, with nomadic populations facing extreme challenges (83.28% unimproved), followed by rural areas (51.10%), in contrast to urban areas (23.88% unimproved). Regional disparities are also highly significant, with a chi-squared value of 2600 and a p-value less than 0.001, ranging from Hiraan's alarming 90.65% unimproved facilities to Lower Shabelle's mere 3.70%, underscoring the necessity for context-specific and targeted interventions to address these diverse challenges across Somalia.

Table 1 Descriptive Statistics of Unimproved Sanitation Facilities in Somalia

**Variable**<br>*Individual-level variables*	**Categories**	**Sanitation Facilities**		**Chi2**	**P-value**
		Unimproved (%)	Improved (%)		
**Sanitation Facility**		36.87	63.13		
**Age of household head**	<20 years	32.74	67.26	47.3653	0.001
	20-29 years	34.71	65.29		
	30-39 years	38.97	61.03		

	40-49 years	40.45	59.55		
	50-59 years	41.59	58.41		
	60+ years	37.64	62.36		
**Sex of household head**	Male	38.58	61.42	12.7784	0.001
	Female	35.42	64.58		
**Ever Attended school**	Yes	30.12	69.88	247.5964	0.001
	No	44.02	55.98		
**Marital Status**	Married	42.66	57.34	153.5151	0.001
	Divorced	31.27	68.73		
	Never married	31.51	68.49		
	Widowed	33.58	66.42		
**Housing type category**	Temporary/Informal/Basic	61.32	38.68	1,300	0.001
	Permanent/Formal	26.79	73.21		
**Lighting-source**	Traditional/Other	58.86	41.14	1,200	0.001
	Modern/Improved	26.15	73.85		
**Household Ownership**	Yes	36.53	63.47	4.7038	0.030
	No,non-household member	39.56	60.44		
**IDP**	Yes	64.27	35.73	173.5672	0.001
	No	35.64	64.36		
**Use Internet**	Yes	21.62	78.38	691.7784	0.001
	No	45.71	54.29		
**Electricity Access**	Yes	23.12	76.88	1,600	0.001
	No	59.58	40.42		
**Labour_status**	Yes	39.04	60.96	3.6606	0.056
	No	36.54	63.46		
**Food security**	Food Secure	25.17	74.83	593.9134	0.001
	Food Insecure	46.76	53.24		
Community Level Variables					
**Type of Residence**	Rural	51.10	48.90	1,800	0.001
	Urban	23.88	76.12		
	Nomadic	83.28	16.72		
**Region**	Awdal	44.13	55.87	2,600	0.001
	Bakool	42.61	57.39		
	Banadir	5.37	94.63		
	Bari	11.38	88.62		
	Bay	80.39	19.61		
	Galgaduud	36.57	63.43		
	Gedo	21.27	78.73		
	Hiraan	90.65	9.35		
	Lower Juba	53.09	46.91		
	Lower Shabelle	3.70	96.30		
	Waqooyi Galbeed	41.67	58.33		
	Middle Shabelle	18.68	81.32		
	Mudug	29.01	70.99		
	Nugaal	52.02	47.98		
	Sanaag	50.54	49.46		
	Sool	27.62	72.38		
	Togdheer	27.78	72.22		

3.2 Multilevel Logistic Regression

The multilevel analysis of unimproved sanitation in Somalia reveals that both individual and community-level factors are significant determinants as depicted in Table 2. The initial empty model (model 0) established the variance justifying a multilevel approach. Model 1, focusing on individual factors, identified that permanent/formal housing (AOR: 3.67) and IDP status (AOR: 3.37) substantially increase the odds of unimproved sanitation, highlighting paradoxes in housing quality and the severe vulnerability of displaced populations. Conversely, not being a household member, not using the internet, lacking electricity, and food insecurity were all associated with lower odds of unimproved sanitation, suggesting complex interdependencies between modernization, socio-economic status, and access to basic services.

Model 2, examining community factors, found that urban residence dramatically elevated the odds of unimproved sanitation (AOR: 14.46), indicating that rapid urbanization outpaces infrastructure development. In contrast, nomadic populations showed a remarkably low likelihood (AOR: 0.01), likely due to their reliance on open defecation. Regional disparities were extreme, with Lower Shabelle exhibiting an exceptionally high AOR (213.19), while regions like Hiraan and Bay had significantly lower odds, underscoring profound geographical inequalities.

The full model (Model 3) integrated these findings, showing that while individual factors like permanent housing and IDP status remain critical, community context, particularly urban residence (AOR: 7.99) and the severe regional disparities (e.g., Lower Shabelle AOR: 175.42), are powerful overarching drivers of unimproved sanitation. This comprehensive analysis emphasizes the need for tailored interventions addressing both household vulnerabilities and broader structural and regional inequalities in Somalia.

Table 2. Multilevel Analysis Unimproved Sanitation Facilities in Somalia

Variable	Categories	Model 0: Empty Model (AOR [95% CL])	Model 1: Individual Factors (AOR [95% CL])	Model 2: Community Factors (AOR [95% CL])	Model 3: Full Model (AOR [95% CL])
**Age of household head**	20-29 years		0.83 [0.67, 1.04]		0.84 [0.67,1.05]
	30-39 years		0.82 [0.64, 1.06]		0.81 [0.63,1.04]
	40-49 years		0.94 [0.69, 1.27]		0.92 [0.68,1.25]
	50-59 years		0.83 [0.60, 1.16]		0.81 [0.58,1.13]
	60+ years		0.89 [0.65, 1.20]		0.86 [0.63, 1.16]
**Sex of household head**	Female		1.14 [0.99, 1.31]		1.13 [0.98, 1.30]
**Ever Attended school**	No		1.03 [0.87, 1.21]		1.02 [0.87, 1.20]
**Marital Status**	Divorced		0.90 [0.66, 1.23]		0.88 [0.64, 1.21]
	Never married		0.88 [0.70, 1.09]		0.85 [0.68, 1.06]
	Widowed		1.08 [0.82, 1.43]		1.06 [0.80, 1.40]
**Housing type category**	Permanent/Formal		3.67 [2.97, 4.53]		3.42 [2.76, 4.23]

**Lighting-source category**	Modern/Improved	0.88 [0.65, 1.19]		0.84 [0.62, 1.13]
**Household Ownership**	No, non-household member	0.69 [0.55, 0.86]		0.69 [0.55, 0.87]
**IDP**	No	3.37 [2.16, 5.23]		3.18 [2.06, 4.92]
**Use Internet**	No	0.68 [0.57, 0.80]		0.71 [0.60, 0.84]
**Electricity Access**	No	0.39 [0.28, 0.54]		0.44 [0.32, 0.60]
**Labour_status**	No	1.04 [0.85, 1.28]		1.04 [0.85, 1.28]
**Food security**	Food Insecure	0.63 [0.54, 0.75]		0.66 [0.56, 0.78]
**Type of Residence**	Urban		14.46 [7.70, 27.15]	7.99 [4.35,14.69]
	Nomadic		0.01 [0.004, 0.035]	0.04 [0.01,0.11]
**Region**	Bakool		0.66 [0.14, 3.05]	1.80 [0.41, 7.83]
	Banadir		23.18 [5.05,106.45]	17.12[3.88,75.61]
	Bari		12.37 [2.67, 57.34]	11.64[2.6, 52.6]
	Bay		0.04 [0.01, 0.2]	0.08[0.02, 0.39]
	Galgaduud		1.73 [0.40, 7.44]	2.39 [0.58, 9.82]
	Gedo		6.01 [1.31, 27.46]	11.98 [2.68,53.5]
	Hiraan		0.0009 [0.0001, 0.0051]	0.003[0.001,0.02]
	Lower Juba		0.29 [0.06,1.46]	0.37 [0.08, 1.70]
	Lower Shabelle		213.19 [27.92,1627.65]	175 [24.4,1259.9]
	Waqooyi Galbeed		1.34 [0.35, 5.07]	1.26 [0.35, 4.58]
	Middle Shabelle		7.09 [1.63, 30.78]	12.77 [3.02, 54]
	Mudug		2.64 [0.57, 12.29]	2.94 [0.66, 13.07]
	Nugaal		0.15 [0.03, 0.73]	0.15 [0.03, 0.67]
	Sanaag		0.39 [0.08, 1.80]	0.36 [0.08, 1.57]
	Sool		4.96 [1.03, 23.78]	4.75 [1.05, 21.41]
	Togdheer		14.77 [3.21, 67.87]	17.00 [3.80, 76.1]

3.3 Logistic Regression Models comparison

The models comparison in Table 2 provides a clear progression in model fit and explanation of variance regarding unimproved sanitation facilities in Somalia. The initial Empty Model (Model 0), with a Log-likelihood of -3978.9811, an AIC of 7961.962, and a BIC of 7976.739, reveals a substantial intraclass correlation coefficient (ICC) of 0.9396. This high ICC indicates that approximately 94% of the total variance in unimproved sanitation is attributable to differences between clusters (enumeration areas), underscoring the necessity of a multilevel approach. As individual-level variables are introduced in Model 1, the Log-likelihood improves to -3797.8329, while the AIC (7635.666) and BIC (7783.434) decrease, signifying a better model fit. The ICC also drops to 0.8792, suggesting that individual household characteristics explain some of the variance in sanitation status. Model 2, incorporating only community-level variables, shows an even greater improvement, with a Log-likelihood of -3784.4915 and further reductions in AIC (7608.983) and BIC (7756.751). The ICC in this model is 0.7786, indicating that community-level factors account for a significant portion of the variability in unimproved sanitation. Finally, Model 3, the Full Model, which includes both individual and community-level variables, demonstrates the best fit among

all models. It achieves the highest Log-likelihood of -3640.3183 and the lowest AIC (7356.637) and BIC (7637.396). The ICC in the Full Model is 0.7418, the lowest of all models, implying that the combined individual and community-level factors substantially reduce the unexplained variance between clusters. The consistent decrease in AIC, BIC, and ICC values across the models, culminating in the Full Model, confirms that a multilevel approach integrating both individual and community-level determinants is essential for comprehensively understanding the factors influencing unimproved sanitation facilities in Somalia.

Table 3 Models comparison

Model	N	Log-likelihood	AIC	BIC	ICC
**Model 0 (Empty)**	11,949	-3978.9811	7961.962	7976.739	0.9396
**Model 1 (Individual)**	11,949	-3797.8329	7635.666	7783.434	0.8792
**Model 2 (Community)**	11,949	-3784.4915	7608.983	7756.751	0.7786
**Model 3 (Full)**	11,949	-3640.3183	7356.637	7637.396	0.7418

3.4 Spatial Analysis Result

3.4.1 Spatial distribution

The spatial distribution of unimproved sanitation facilities in Somalia, as depicted in Figure 1, reveals stark regional disparities, with certain areas emerging as critical hotspots while others demonstrate remarkable progress. Hiraan and Bay regions, marked by the darkest red, exhibit an alarmingly high prevalence of unimproved sanitation at 90.65% and 80.39% respectively, indicating severe challenges in access to basic facilities. Other regions like Lower Juba, Nugaal, and Sanaag also show high rates above 50%, represented by darker orange hues. Conversely, Lower Shabelle and Banadir stand out as significant cold spots, with exceptionally low prevalence rates of 3.70% and 5.37%, respectively, suggesting better sanitation infrastructure and access, possibly due to their more urbanized nature or successful localized interventions. Regions such as Bari, Gedo, and Middle Shabelle also report comparatively lower figures, yet still experience moderate challenges. This clear geographical clustering of high and low prevalence areas underscores the urgent need for context-specific and targeted interventions to address the profound inequalities in sanitation access across Somalia.

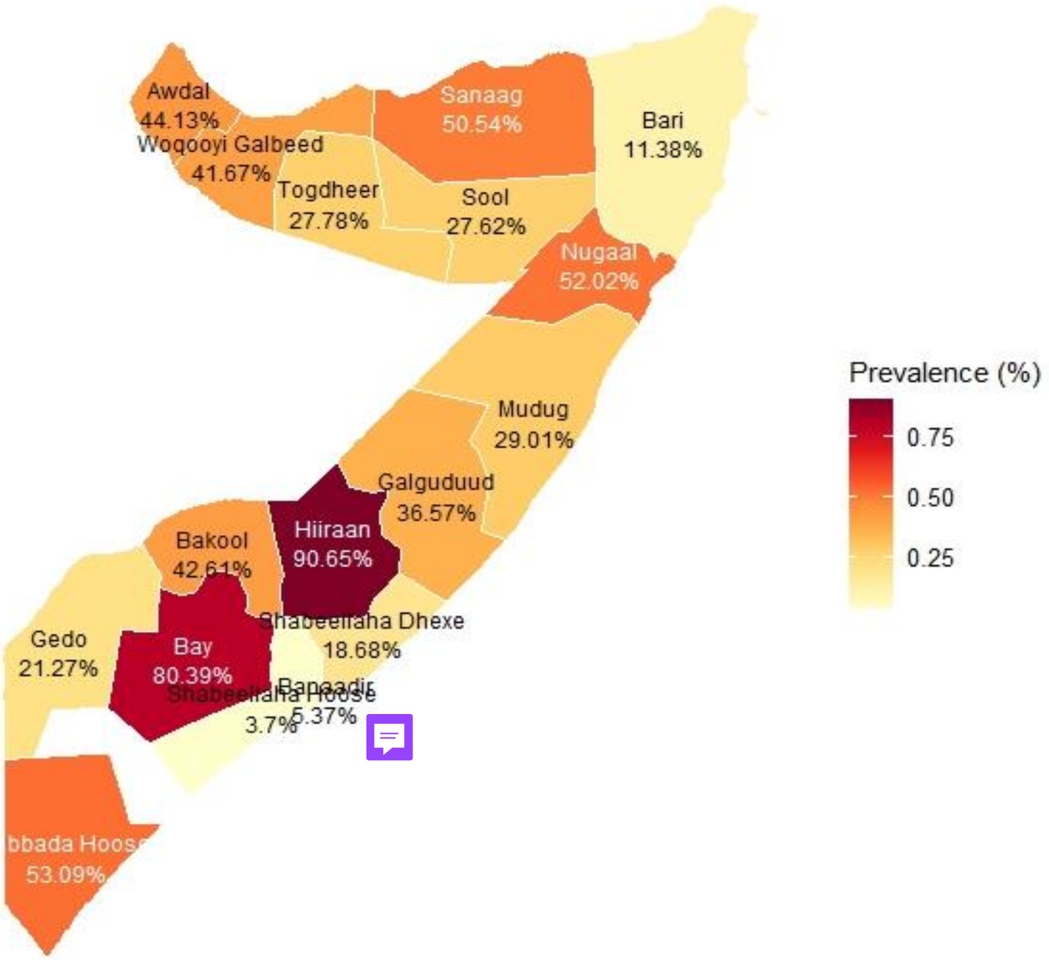

Figure 1 Spatial Distribution of Unimproved Sanitation Facilities

3.4.2 Spatial Autocorrelation-Global Moran's I

Figure 2, the Global Moran's I normal distribution curve, indicates that the spatial distribution of unimproved sanitation facilities in Somalia is not statistically clustered or dispersed. With a Moran's Index of -0.19894, a z-score of -0.75544, and a high p-value of 0.77501, the data suggests a random spatial pattern, meaning there's no significant global clustering of high or low prevalence areas.

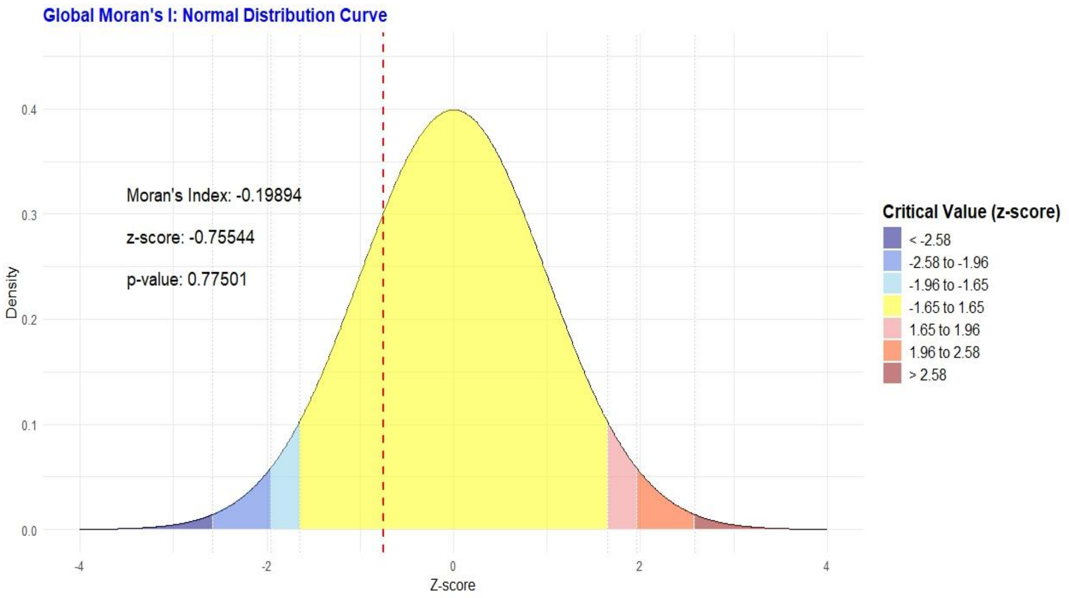

Figure 2 Global Moran's I for spatial Autocorrelation of unimproved sanitation facilities

3.4.3 Spatial autocorrelation-local Moran's I

Figure 3, composed of two sets of maps, illustrates the localized patterns of unimproved sanitation facilities in Somalia using Local Moran's I and corresponding p-values. The maps on the left side (grey with blue patches) identify specific areas where local spatial clustering is statistically significant, meaning the prevalence of unimproved sanitation in those blue regions is not random compared to their neighbors. The maps on the right, which are almost entirely dark blue, strongly indicate that for nearly all regions across Somalia, the observed local spatial patterns of unimproved sanitation are statistically significant (p-value less than 0.05). This means that, despite a lack of overall global clustering, there are distinct and statistically meaningful local concentrations either "hotspots" of high unimproved sanitation or "coldspots" of low unimproved sanitation across the vast majority of the country's administrative divisions.

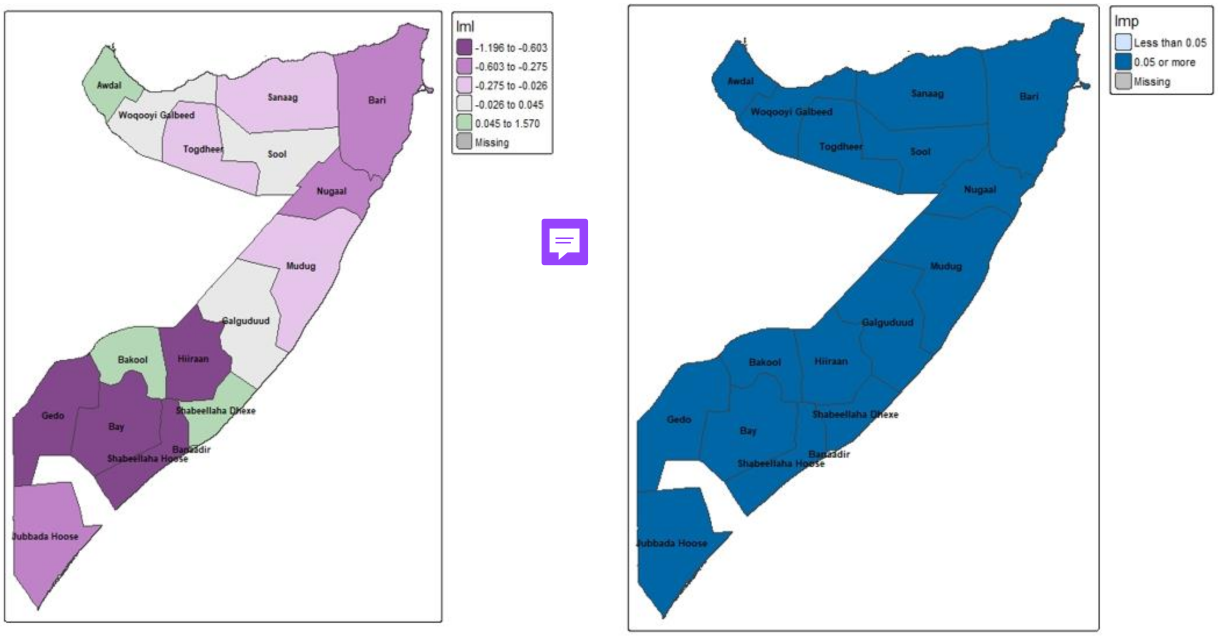

Figure 3 Local Moran's I and its p-value for significance

3.4.4 Hot spot and cold spot analysis

Figure 4, displaying the Getis-Ord Gi* results, maps the spatial distribution of statistically significant hot spots and cold spots of unimproved sanitation facilities across Somalia. The color gradient in the legend indicates the range of Gi* values, which identify areas with high values (hot spots) or low values (cold spots) clustered together. Darker green shades (0.615 to 1.603) represent statistically significant hot spots, indicating regions where high prevalence of unimproved sanitation facilities is clustered. We can observe these hot spots prominently in areas like Hiraan, Bay, Galgaduud, Gedo, Mudug, and parts of the northern regions such as Nugaal. Conversely, the lighter purple shades (-1.847 to -0.979) represent statistically significant cold spots, signifying clusters of low prevalence of unimproved sanitation facilities. These cold spots appear in regions like Awdal, Juba Hoose, and parts of the eastern coast (Bari). Intermediate shades of purple and green suggest areas with less extreme clustering or values closer to the mean. This Getis-Ord Gi* analysis visually corroborates and further refines the understanding of spatial disparities, clearly pinpointing specific regions that are hotbeds of unimproved sanitation and those with better access, thereby providing critical insights for geographically targeted public health interventions.

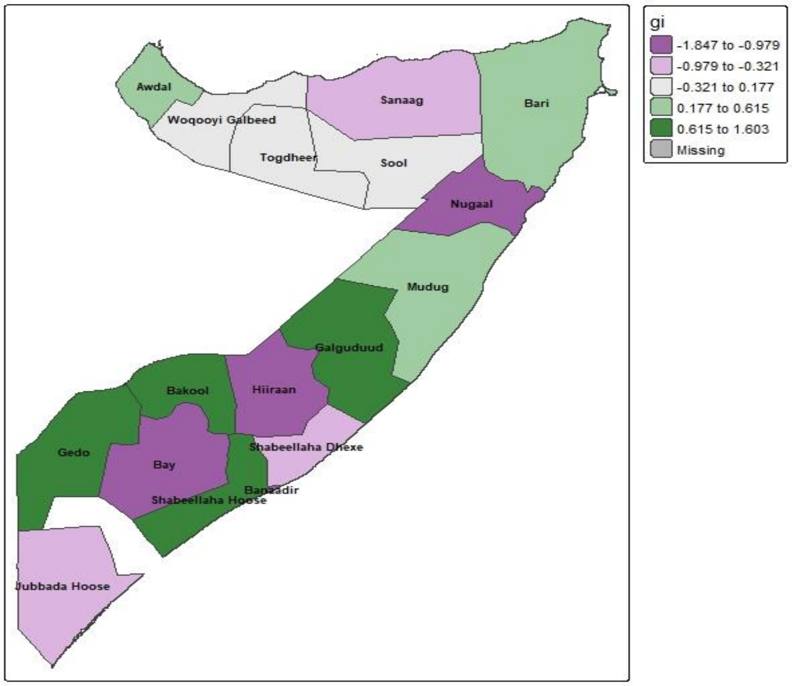

Figure 4 Hot and cold spots region using Getis-Ord G*

4. Discussion

This study's multilevel and spatial analysis provides a granular understanding of the complex determinants driving the use of unimproved sanitation in Somalia, with findings that both corroborate and challenge existing literature. The overall prevalence of over one-third of households using unimproved facilities highlights a persistent public health crisis. This figure is situated within a range of estimates for Somalia, including a 2024 study using 2020 data that reported 22.3% of households using unimproved facilities and an earlier survey indicating a higher rate of 59.7% (Saleem et al., 2019; Unicef, 2018). These variations likely reflect differences in survey methodologies and the dynamic nature of displacement and infrastructure challenges in the country.

A key finding is the stark disparity across residence types, with nomadic populations exhibiting an overwhelming 83.28% prevalence of unimproved sanitation. This is consistent with the understanding that nomadic lifestyles present significant challenges for fixed sanitation infrastructure, often leading to a reliance on open defecation (Demoze et al., 2025; Pullan et al., 2014). The significantly lower odds of unimproved sanitation among nomadic groups in the multilevel model (AOR: 0.04) is a statistical reflection of open defecation not being classified as a *facility*, rather than indicating better access. In contrast, the high odds for urban residents (AOR:

7.99) compared to their rural counterparts points to a crisis of rapid, unplanned urbanization outstripping the provision of basic services, a phenomenon observed in other rapidly growing urban centers in sub-Saharan Africa. A recent study in Ethiopia, for example, found that while urban areas overall had better access, certain urban populations were highly vulnerable (J. Brown et al., 2013; Yusuf et al., 2023c).

The multilevel analysis revealed several nuanced and seemingly paradoxical associations. The finding that households in permanent/formal housing had significantly higher odds of using unimproved sanitation (AOR: 3.42) is counterintuitive but may be explained by the context of protracted displacement. Many internally displaced persons (IDPs) live in camps where the structures are considered "permanent," yet they lack adequate sanitation services (Dahir et al., 2024; Spears et al., 2013). This is strongly supported by the finding that IDP status itself dramatically increases the odds of using unimproved sanitation by more than threefold (AOR: 3.18), confirming the extreme vulnerability of this population group, a fact well-documented in humanitarian reports on Somalia (Dahir et al., 2024; ESCAP, 2016; Spears et al., 2013)[.

The spatial analysis was instrumental in moving beyond national averages to identify specific geographic inequities. The identification of Hiraan (90.65%) and Bay (80.39%) as critical hotspots aligns with the known impacts of conflict, climate shocks, and instability on these regions, which severely impede infrastructure development. Conversely, the significantly better outcomes in the more urbanized regions of Banadir (5.37%) and Lower Shabelle (3.70%) underscore the importance of governance and the presence of humanitarian and development actors. The Getis-Ord Gi* analysis, by confirming these statistically significant hot and cold spots, provides a clear evidence base for geographically targeted interventions. While the Global Moran's I indicated no overall global clustering, the significant local clustering reinforces the idea that the drivers of poor sanitation are highly localized. This aligns with broader research across sub-Saharan Africa, which increasingly calls for context-specific, decentralized approaches to address sanitation challenges effectively (Azanaw et al., 2023; Hopewell & Graham, 2014; Osumanu et al., 2019).

5. Conclusion

This study reveals that the state of sanitation in Somalia is characterized by deep inequalities driven by a combination of socioeconomic vulnerability, geographic location, and lifestyle. The prevalence of unimproved sanitation is unacceptably high, particularly among nomadic populations, rural communities, and internally displaced persons. Individual-level factors such as housing type and displacement status, and community-level factors like place of residence and

region, are powerful determinants of sanitation access. The spatial analysis underscores that the burden of poor sanitation is not evenly distributed, with specific regions like Hiraan and Bay facing a critical public health emergency. This situation poses severe risks to public health, dignity, and safety, particularly for women and girls.

The findings strongly advocate for a multi-pronged approach to address Somalia's sanitation crisis. Interventions must be geographically targeted, prioritizing the identified hotspots with urgent investment in basic sanitation infrastructure and services. Furthermore, policies must be tailored to the distinct needs of urban, rural, and nomadic populations, with a special focus on the highly vulnerable IDP communities. Addressing the underlying drivers identified in the multilevel analysis, such as the paradox of formal housing with poor services, requires integrated development planning that ensures housing projects are accompanied by essential services. Ultimately, improving sanitation in Somalia is not merely an infrastructure challenge but a critical component of public health, human dignity, and sustainable development that demands immediate and evidence-informed action from the government and its international partners, in line with the Sustainable Development Goals' aim to achieve universal access to sanitation for all.

Reference

Ahmed, H. A., & Ali, D. A. (2024). Prevalence and determinants of household access to improved latrine utilization in Somalia: health demographic survey (SHDS) 2020. *Environmental Health Insights*, *18*, 11786302241284148.

Azanaw, J., Abera, E., Malede, A., & Endalew, M. (2023). A multilevel analysis of improved drinking water sources and sanitation facilities in Ethiopia: Using 2019 Ethiopia mini demographic and health survey. *Frontiers in Public Health*, *11*, 1063052.

Belay, D. G., Asratie, M. H., Aragaw, F. M., Tsega, N. T., Endalew, M., & Gashaw, M. (2022). Open defecation practice and its determinants among households in sub-Saharan Africa: pooled prevalence and multilevel analysis of 33 sub-Saharan Africa countries demographic and health survey. *Tropical Medicine and Health*, *50*(1), 28.

Brown, C., Neves-Silva, P., & Heller, L. (2016). The human right to water and sanitation: a new perspective for public policies. *Ciencia & Saude Coletiva*, *21*, 661–670.

Brown, J., Cairncross, S., & Ensink, J. H. J. (2013). Water, sanitation, hygiene and enteric infections in children. *Archives of Disease in Childhood*, *98*(8), 629–634.

Dahir, H. G., Ahmed, A. T., & Abdilahi, M. A. (2024). *Burden and Determinants of Child Mortality in Somalia analysis of SDHS 2020*.

Demoze, L., Tigabie, M., Worede, E. A., Brhan, H., Gizaw, Z., Tesfaye, A. H., & Yitageasu, G. (2025). Determinants and geographic distribution of unimproved sanitation facilities in sub-Saharan Africa, spatial and multilevel analysis using demographic and health survey (DHS) data. *BMC Public Health*, *25*(1), 2848.

Demsash, A. W., Tegegne, M. D., Wubante, S. M., Walle, A. D., Donacho, D. O., Senishaw, A. F., Emanu, M. D., & Melaku, M. S. (2023). Spatial and multilevel analysis of sanitation service access and related factors among households in Ethiopia: using 2019 Ethiopian national dataset. *PLOS Global Public Health*, *3*(4), e0001752.

Desye, B., Geto, A. K., Daba, C., Bezie, A. E., Reda, S., Sisay, T., Getachew, M., & Berhanu, L. (2025). Spatial and multilevel analysis of determinant factors for safely managed sanitation services in Ethiopia. *Scientific Reports*, *15*(1), 31027.

ESCAP, U. N. (2016). *Sustainable Development Goal 6: ensure availability and sustainable management of water and sanitation for all*.

Heijnen, M., Cumming, O., Peletz, R., Chan, G. K.-S., Brown, J., Baker, K., & Clasen, T. (2014). Shared sanitation versus individual household latrines: a systematic review of health outcomes. *PloS One*, *9*(4), e93300.

Hopewell, M. R., & Graham, J. P. (2014). Trends in access to water supply and sanitation in 31 major sub-Saharan African cities: an analysis of DHS data from 2000 to 2012. *BMC Public Health*, *14*(1), 208.

Novotný, J., & Mamo, B. G. (2022). Household - level sanitation in Ethiopia and its influencing factors : a systematic review. *BMC Public Health*, 1–16. https://doi.org/10.1186/s12889-022-13822-5

Organization, W. H. (2022). *Country cooperation strategy for WHO and Somalia 2021–2025*.

Osumanu, I. K., Kosoe, E. A., & Ategeeng, F. (2019). Determinants of open defecation in the Wa municipality of Ghana: empirical findings highlighting sociocultural and economic dynamics among households. *Journal of Environmental and Public Health*, *2019*(1), 3075840.

Pullan, R. L., Freeman, M. C., Gething, P. W., & Brooker, S. J. (2014). Geographical inequalities in use of improved drinking water supply and sanitation across sub-Saharan Africa: mapping

and spatial analysis of cross-sectional survey data. *PLoS Medicine*, *11*(4), e1001626.

Saleem, M., Burdett, T., & Heaslip, V. (2019). Health and social impacts of open defecation on women: a systematic review. *BMC Public Health*, *19*, 1–12.

Spears, D., Ghosh, A., & Cumming, O. (2013). Open defecation and childhood stunting in India: an ecological analysis of new data from 112 districts. *PloS One*, *8*(9), e73784.

Unicef. (2018). *Drinking water, sanitation and hygiene in schools: global baseline report 2018*.

Yusuf, A. M., Pasukphun, N., & Suma, Y. (2023a). Factors associated with sanitation practices among household-level in Mogadishu, Somalia. *International Journal of Public Health Asia Pacific*, *August*. https://doi.org/10.62992/ijphap.v2i2.24

Yusuf, A. M., Pasukphun, N., & Suma, Y. (2023b). Factors associated with sanitation practices among household-level in Mogadishu, Somalia. *International Journal of Public Health Asia Pacific*.

Yusuf, A. M., Pasukphun, N., & Suma, Y. (2023c). Factors associated with sanitation practices among household-level in Mogadishu, Somalia. *International Journal of Public Health Asia Pacific*, *2*(02), 1–12. https://doi.org/10.62992/ijphap.v2i2.24
