# Supplementary material for: Spatial distribution and determinants of unimproved sanitation facilities among households in Somalia: Using Somalia integrated household budget survey (SIHBS 2022)
Source: PLoS One. 2026 Apr 24;21(4):e0348029. doi: 10.1371/journal.pone.0348029 (PMC13108892; doi:10.1371/journal.pone.0348029)
Supplement: Attachment — Submitted filename: PONE-D-25-58376_R1-AF.pdf [file pone.0348029.s004.pdf]

PLOS One

Spatial Distribution and Determinants of Unimproved Sanitation Facilities Among Households in Somalia: Using Somalia Integrated Household Budget Survey (SIHBS 2022)

--Manuscript Draft--

**Manuscript Number:**	PONE-D-25-58376R1
**Article Type:**	Research Article
**Full Title:**	Spatial Distribution and Determinants of Unimproved Sanitation Facilities Among Households in Somalia: Using Somalia Integrated Household Budget Survey (SIHBS 2022)
**Short Title:**	Spatial Distribution and Determinants of Unimproved Sanitation Facilities Among Households in Somalia
**Corresponding Author:**	Omar Muhumed Maidhane, M.D<br>Amoud University<br>Galkayo, Galmudug SOMALIA
**Keywords:**	Keywords: Unimproved sanitation; Spatial analysis; Multilevel modeling; Somalia; Public health disparities
**Abstract:**	Abstract<br>Background: Access to adequate sanitation remains a critical public health challenge in Somalia, where a large portion of the population relies on unimproved facilities due to persistent conflict, climate shocks, and political instability. This reliance contributes to a high burden of waterborne diseases. This study aimed to assess the spatial distribution of unimproved sanitation and identify its individual and community-level determinants using recent national data to inform targeted interventions.<br>Methods: This study is a secondary analysis of the 2022 Somalia Integrated Household Budget Survey (SIHBS), which included 7,212 households. The primary outcome was the use of unimproved sanitation facilities, categorized according to the WHO/UNICEF Joint Monitoring Programme (JMP) definitions. We employed a multilevel logistic regression model to identify individual and community-level determinants associated with unimproved sanitation. To analyze the spatial patterns of unimproved sanitation, we used Global Moran's I for spatial autocorrelation and the Getis-Ord Gi* statistic for hotspot analysis.<br>Result: Overall, 36.87% of Somali households use unimproved sanitation facilities. There are significant disparities across residence types, with the highest prevalence among nomadic populations (83.28%), followed by rural (51.10%) and urban (23.88%) residents. The multilevel analysis revealed that households in permanent/formal housing (AOR: 3.42) and those with IDP status (AOR: 3.18) had significantly higher odds of using unimproved sanitation. At the community level, urban residence was paradoxically associatedwith higher odds of unimproved sanitation (AOR: 7.99) compared to rural areas, while nomadic populations had significantly lower odds (AOR: 0.04), likely reflecting a high prevalence of open defecation not captured as a "facility."<br>Spatial analysis identified significant hotspots of unimproved sanitation in the Hiraan (90.65%) and Bay (80.39%) regions, and coldspots in Banadir (5.37%) and Lower Shabelle (3.70%).<br>Conclusion: The findings highlight deep inequalities in sanitation access across Somalia, driven by geographic location, socioeconomic status, and population group. The high prevalence of unimproved sanitation, especially among nomadic, rural, and displaced populations, calls for urgent, geographically-targeted interventions. A multi-pronged approach is necessary, focusing on the specific needs of different communities and addressing the underlying structural and individual-level drivers of poor sanitation to advance public health and sustainable development goals in the region.
**Order of Authors:**	Omar Muhumed Maidhane, M.D
	Omran Salih, PhD
	Abdisalan Hassan Muse, PhD

Powered by Editorial Manager® and ProduXion Manager® from Aries Systems Corporation

	Abdirahman Omer Osman
	Muse H Abdi, M.D
	Mahdi Hashi Hassan
	Nur Mohamud Ali
	Shacban Abdillahi Elmi
**Opposed Reviewers:**	
**Response to Reviewers:**	Dear Dr. Alfredo Luis Fort,<br>Thank you for the opportunity to revise our manuscript, "Spatial Distribution and Determinants of Unimproved Sanitation Facilities Among Households in Somalia: Using Somalia Integrated Household Budget Survey (SIHBS 2022)" (PONE-D-25-58376). We are grateful to you and the reviewers for the insightful and constructive feedback, which has significantly improved the quality of our paper
**Additional Information:**	
**Question**	**Response**
**Financial Disclosure**<br><br>Enter a financial disclosure statement that describes the sources of funding for the work included in this submission. Review the submission guidelines for detailed requirements. View published research articles from *PLOS ONE* for specific examples.<br><br>This statement is required for submission and **will appear in the published article** if the submission is accepted. Please make sure it is accurate.<br><br>**Funded studies**<br>Enter a statement with the following details:<br>• Initials of the authors who received each award<br>• Grant numbers awarded to each author<br>• The full name of each funder<br>• URL of each funder website<br>• Did the sponsors or funders play any role in the study design, data collection and analysis, decision to publish, or preparation of the manuscript?<br><br>Did you receive funding for this work?	The author(s) received no specific funding for this work.
**Competing Interests**<br><br>Use the instructions below to enter a competing interest statement for this submission. On behalf of all authors,	The authors have declared that no competing interests

disclose any competing interests that could be perceived to bias this work—acknowledging all financial support and any other relevant financial or non-financial competing interests.<br><br>This statement is required for submission and **will appear in the published article** if the submission is accepted. Please make sure it is accurate and that any funding sources listed in your Funding Information later in the submission form are also declared in your Financial Disclosure statement.<br><br>View published research articles from *PLOS ONE* for specific examples.<br><br>**NO authors have competing interests**<br><br>Enter: *The authors have declared that no competing interests exist.*<br><br>**Authors with competing interests**<br><br>Enter competing interest details beginning with this statement:<br><br>*I have read the journal's policy and the authors of this manuscript have the following competing interests: [insert competing interests here]*<br><br>* typeset	
**Ethics Statement**<br><br>Enter an ethics statement for this submission. This statement is required if the study involved:<br><br>• Human participants<br>• Human specimens or tissue<br>• Vertebrate animals or cephalopods<br>• Vertebrate embryos or tissues<br>• Field research<br><br>Write "N/A" if the submission does not require an ethics statement.	No

General guidance is provided below. Consult the submission guidelines for detailed instructions. **Make sure that all information entered here is included in the Methods section of the manuscript.**<br><br>**Format for specific study types**<br><br>**Human Subject Research (involving human participants and/or tissue)**<br>• Give the name of the institutional review board or ethics committee that approved the study<br>• Include the approval number and/or a statement indicating approval of this research<br>• Indicate the form of consent obtained (written/oral) or the reason that consent was not obtained (e.g. the data were analyzed anonymously)<br><br>**Animal Research (involving vertebrate animals, embryos or tissues)**<br>• Provide the name of the Institutional Animal Care and Use Committee (IACUC) or other relevant ethics board that reviewed the study protocol, and indicate whether they approved this research or granted a formal waiver of ethical approval<br>• Include an approval number if one was obtained<br>• If the study involved *non-human primates*, add *additional details* about animal welfare and steps taken to ameliorate suffering<br>• If anesthesia, euthanasia, or any kind of animal sacrifice is part of the study, include briefly which substances and/or methods were applied<br><br>**Field Research**<br><br>Include the following details if this study involves the collection of plant, animal, or other materials from a natural setting:<br>• Field permit number<br>• Name of the institution or relevant body that granted permission	
**Data Availability**<br><br>Authors are required to make all data	No - some restrictions will apply

underlying the findings described fully available, without restriction, and from the time of publication. PLOS allows rare exceptions to address legal and ethical concerns. See the PLOS Data Policy and FAQ for detailed information.<br><br>A Data Availability Statement describing where the data can be found is required at submission. Your answers to this question constitute the Data Availability Statement and **will be published in the article**, if accepted.<br><br>**Important:** Stating 'data available on request from the author' is not sufficient. If your data are only available upon request, select 'No' for the first question and explain your exceptional situation in the text box.<br><br>Do the authors confirm that all data underlying the findings described in their manuscript are fully available without restriction?	
**Describe where the data may be found in full sentences. If you are copying our sample text, replace any instances of XXX with the appropriate details.**<br><br>• If the data are **held or will be held in a public repository**, include URLs, accession numbers or DOIs. If this information will only be available after acceptance, indicate this by ticking the box below. For example: *All XXX files are available from the XXX database (accession number(s) XXX, XXX.).*<br>• If the data are all contained **within the manuscript and/or Supporting Information files**, enter the following: *All relevant data are within the manuscript and its Supporting Information files.*<br>• If neither of these applies but you are able to provide **details of access elsewhere**, with or without limitations, please do so. For example:<br><br>*Data cannot be shared publicly because of [XXX]. Data are available from the*	The data underlying the results presented in the study are available from the Somali National Bureau of Statistics (SNBS).[1][2][3] The dataset, the Somalia Integrated Household Budget Survey (SIHBS) 2022, can be requested by researchers through the Somali National Data Archive (SoNADA) portal on the SNBS website.[4]

XXX Institutional Data Access / Ethics Committee (contact via XXX) for researchers who meet the criteria for access to confidential data.<br><br>*The data underlying the results presented in the study are available from (include the name of the third party and contact information or URL).*<br>• This text is appropriate if the data are owned by a third party and authors do not have permission to share the data.<br><br>* typeset	
Additional data availability information:	

Powered by Editorial Manager® and ProduXion Manager® from Aries Systems Corporation

Cover Letter

Omran Salih
Amoud University
omran.salih@amoud.edu.so

Date: 27/10/2025

To: Editor-in-Chief
PLOS One

Dear Editor,

I am pleased to submit our original research manuscript entitled *"Spatial Distribution and Determinants of Unimproved Sanitation Facilities Among Households in Somalia: Using Somalia Integrated Household Budget Survey (SIHBS 2022)"* for consideration for publication in PLOS One Journal.

This study addresses a pressing public health concern in Somalia, inadequate sanitation by employing both multilevel and geospatial analytical approaches. Using nationally representative data from the SIHBS 2022, we examined the spatial distribution and determinants of unimproved sanitation facilities across Somali households. The analysis identified substantial geographic disparities and highlighted vulnerable population groups, including nomadic and internally displaced communities, who are disproportionately affected.

Our findings contribute to the limited body of evidence on sanitation inequalities in fragile and conflict-affected settings. The study provides actionable insights for policymakers and development partners to design geographically targeted and context-specific interventions aimed at achieving Sustainable Development Goal 6 (Clean Water and Sanitation).

We believe this manuscript aligns well with the journal's scope and audience, as it provides novel evidence linking spatial, social, and environmental determinants of health outcomes in low-resource and conflict-affected contexts. The manuscript is original, has not been published elsewhere, and is not under consideration by any other journal.

We sincerely hope that you find this study valuable and suitable for publication in PLOS One journal. Thank you for considering our submission. We look forward to your positive response.

Sincerely,
Omar

On behalf of all co-authors

Manuscript Click here to access/download;Manuscript;Manuscript.docx

Spatial Distribution and Determinants of Unimproved Sanitation Facilities Among Households in Somalia: Using Somalia Integrated Household Budget Survey (SIHBS 2022)

Omar Muhumed Maidhane[1,3], Omran Salih[1,4,*], Abdisalam Hassan Muse[1,2], Abdirahman Omer Osman[1], Muse H Abdi[1], Mahdi Hashi Hassan[1], Nur Mohamud Ali[1], Shacban Abdilahi Elmi[1]

[1]School of Postgraduate Studies and Research, Amoud University, Borama 25263, Somalia

[2]Research and Innovation Center, Amoud University, Borama 25263, Somalia

[3]Research and Innovation Center, Galkayo University, Galmudug, Somalia.

[4]Institute of System Science, Durban University of Technology, Durban, South Africa

[*]Corresponding Author: *Omar Muhumed Maidhane*

Abstract

Background: Access to adequate sanitation remains a critical public health challenge in Somalia, where a large portion of the population relies on unimproved facilities due to persistent conflict, climate shocks, and political instability. This reliance contributes to a high burden of waterborne diseases. This study aimed to assess the spatial distribution of unimproved sanitation and identify its individual and community-level determinants using recent national data to inform targeted interventions.

Methods: This study is a secondary analysis of the 2022 Somalia Integrated Household Budget Survey (SIHBS), which included 7,212 households. The primary outcome was the use of unimproved sanitation facilities, categorized according to the WHO/UNICEF Joint Monitoring Programme (JMP) definitions. We employed a multilevel logistic regression model to identify individual and community-level determinants associated with unimproved sanitation. To analyze the spatial patterns of unimproved sanitation, we used Global Moran's I for spatial autocorrelation and the Getis-Ord Gi* statistic for hotspot analysis.

Results: Overall, 36.87% of Somali households use unimproved sanitation facilities. There are significant disparities across residence types, with the highest prevalence among nomadic populations (83.28%), followed by rural (51.10%) and urban (23.88%) residents. The multilevel analysis revealed that households in permanent/formal housing (AOR: 3.42) and those with IDP status (AOR: 3.18) had significantly higher odds of using unimproved sanitation. At the community level, urban residence was paradoxically associated with higher odds of unimproved sanitation (AOR: 7.99) compared to rural areas, while nomadic populations had significantly lower odds (AOR: 0.04), likely reflecting a high prevalence of open defecation not captured as a "facility." Spatial analysis identified significant hotspots of unimproved sanitation in the Hiraan (90.65%) and Bay (80.39%) regions, and cold spots in Banadir (5.37%) and Lower Shabelle (3.70%).

Conclusion: The findings highlight deep inequalities in sanitation access across Somalia, driven by geographic location, socioeconomic status, and population group. The high prevalence of unimproved sanitation, especially among nomadic, rural, and displaced populations, calls for urgent, geographically-targeted interventions. A multi-pronged approach is necessary, focusing on the specific needs of different communities and addressing the underlying structural and individual-level drivers of poor sanitation to advance public health and sustainable development goals in the region.

Keywords: Unimproved sanitation; Spatial analysis; Multilevel modeling; Somalia; Public health disparities

1. Introduction

Access to adequate and equitable sanitation is a fundamental human right and a critical component of public health and sustainable development (Yusuf et al. 2023a). Globally, 3.5 billion people still lacked safely managed sanitation in 2022, with 419 million practicing open defecation (Saleem et al., 2019). The disease burden resulting from inadequate sanitation is immense, contributing to the transmission of diseases like cholera, typhoid, and intestinal worm infections, which disproportionately affect children under five (Demoze et al., 2025; Unicef, 2018). Poor sanitation is a significant factor in environmental degradation and is closely linked to childhood stunting and malnutrition (Demoze et al., 2025; Pullan et al., 2014). The Sustainable Development Goals (SDGs), particularly Target 6.2, aim to achieve universal access to sanitation for all and end open defecation by 2030, paying special attention to the needs of women, girls, and those in vulnerable situations (Yusuf et al., 2023b).

Sub-Saharan Africa faces the most significant challenges in sanitation coverage, with disparities between urban and rural areas and slow overall progress (J. Brown et al., 2013; Spears et al., 2013). Somalia, in particular, confronts acute sanitation challenges exacerbated by decades of conflict, political instability, climate-related disasters such as recurrent droughts, and large-scale displacement (Ahmed & Ali, 2024; Yusuf et al., 2023b). According to the WHO/UNICEF Joint Monitoring Programme (JMP), access to even basic sanitation facilities in Somalia is critically low, with a significant portion of the population, especially in rural and nomadic communities, resorting to unimproved facilities or open defecation (ESCAP, 2016; Hopewell & Graham, 2014). Recent data from the 2020 Somalia Demographic Health and Survey indicated that a substantial proportion (59.7%) of households utilized unimproved sanitation facilities (Azanaw et al., 2023). This situation poses severe risks to public health, dignity, and safety, particularly for women and girls who face heightened risks of harassment and violence when seeking private places to relieve themselves (Osumanu et al., 2019).

Despite the clear urgency, there is limited comprehensive research on the specific factors driving the prevalence of unimproved sanitation in Somalia. While several studies have highlighted the scale of the problem, a detailed analysis that combines both spatial and multilevel approaches to understand the geographic disparities and the interplay of individual and community-level determinants is lacking (Belay et al., 2022; Unicef, 2018). Such an analysis is crucial for designing evidence-based, targeted interventions that can address the root causes of poor sanitation. Therefore, this study aims to assess the spatial distribution and identify the determinants of unimproved sanitation facilities among households in Somalia, using recent data from the Somalia Integrated Household Budget Survey (SIHBS 2022).

This study provides a novel and in-depth analysis of sanitation challenges in Somalia by being one of the first to combine a multilevel modeling approach with advanced spatial analysis. While previous research has acknowledged the poor state of sanitation in the country, this study moves beyond descriptive statistics to simultaneously investigate both individual and community-level determinants of unimproved sanitation. The use of the recent and comprehensive 2022 Somalia Integrated Household Budget Survey (SIHBS) dataset allows for a more current and granular understanding of the issue. Furthermore, the application of geospatial techniques such as Getis-Ord Gi* hotspot analysis and spatial autocorrelation provides a clear visual and statistical identification of geographic disparities, pinpointing specific regions and communities that are most affected. This dual approach offers a more robust evidence base for policymakers and public health stakeholders, enabling the design of targeted, context-specific interventions that address both the socioeconomic drivers and the geographical clustering of poor sanitation in Somalia.

2. Material And Methods

2.1 Study Area

The study was conducted in Somalia, a country in the Horn of Africa with an estimated 2024 population of around 19 million and a low population density of approximately 32 people per square kilometer. The nation's economy is primarily agrarian, with the livestock sector accounting for a significant portion of the GDP and over half of export earnings. Despite recent economic growth, Somalia is classified as a least-developed country, and in 2022, over half of the population lived below the national poverty line. Decades of civil conflict and severe climate shocks have devastated public infrastructure, including water and sanitation systems, leading to widespread displacement and vulnerability among its diverse urban, rural, and nomadic communities(Yusuf et al., 2023b).

2.2 Study Design and Data Source

This study employed a secondary analysis of data from the 2022 Somalia Integrated Household Budget Survey (SIHBS), a nationally representative, community-based cross-sectional survey. The SIHBS was conducted by the Somalia National Bureau of Statistics (SNBS) and is the first comprehensive household budget survey in the country since 1985 (Demsash et al., 2023).

The survey used a two-stage stratified cluster sampling design, covering urban, rural, and nomadic populations. In the first stage, enumeration areas (EAs) were selected with probability proportional to their size, followed by the systematic selection of a fixed number of households from each EA in the second stage. The survey included a total sample of 7,212 households. This analysis utilized the final weighted sample to ensure national representativeness.

2.3 Data validation

To ensure the findings are representative of the national population and to account for the complex survey design, sampling weights provided in the SIHBS dataset were applied during all statistical analyses. This weighting procedure adjusts for the unequal probability of selection and non-response rates, ensuring reliable and valid estimates.

2.4 Study variables

The primary outcome variable was the status of the household sanitation facility, categorized as a binary variable: "unimproved" or "improved." This classification was based on the WHO/UNICEF JMP definitions (Organization, 2022). "Unimproved sanitation facilities" were defined as pit latrines without a slab or platform, hanging latrines, bucket latrines, and the practice of open defecation (Brown et al., 2016). *This was coded as '1'.* "Improved sanitation facilities" included flush or pour-flush toilets connected to a piped sewer system, septic tanks, or pit latrines; ventilated improved pit latrines; and pit latrines with a slab, which are not shared with other households. *This was coded as '0'.*

The independent variables were classified into individual-level and community-level factors. Individual-level variables included the sex of the household head (male or female), age of the household head (categorized), educational attainment (no education, primary, secondary, or higher), household size, and the household wealth index, which was categorized into five quintiles: poorest, poorer, middle, richer, and richest. Community-level variables comprised the place of residence (urban, rural, or nomadic), region or federal member state, and community

media exposure, defined as the proportion of households within the enumeration area (EA) that had access to radio or television.

2.6 Data Analysis

2.6.1 Univariant and Multilevel Analysis

Data cleaning, coding, and analysis were performed using STATA version 17. Descriptive statistics, including frequencies and percentages, were used to summarize the characteristics of the study population and the prevalence of unimproved sanitation. Due to the hierarchical nature of the survey data, where households (level 1) are nested within enumeration areas or clusters (level 2), a multilevel logistic regression model was employed to identify the determinants of unimproved sanitation (Desye et al., 2025). This approach accounts for the clustering effect and avoids the underestimation of standard errors that can occur with standard logistic regression (Demoze et al., 2025; Heijnen et al., 2014).

Four consecutive models were fitted: a null model (Model 0) with no explanatory variables to assess the variance in unimproved sanitation between clusters; Model I included only individual-level variables; Model II included only community-level variables; and Model III, the final model, included both individual and community-level variables simultaneously. Measures of variation (random effects) such as the Intraclass Correlation Coefficient (ICC), Median Odds Ratio (MOR), and Proportional Change in Variance (PCV) were calculated to quantify the extent of clustering. Model fit was assessed using the Akaike information criterion (AIC) and Bayesian Information criterion (BIC), with the model having the lowest AIC and BIC values considered the best fit. Adjusted Odds Ratios (AOR) with 95% confidence intervals (CI) were used to report the final associations, and a p-value of < 0.05 was considered statistically significant.

2.6.2 Spatial Analysis

Geospatial analysis was conducted to explore the spatial distribution and identify geographic hotspots of unimproved sanitation facilities in Somalia using ArcGIS version 10.7 and SaTScan version 10.1. First, spatial autocorrelation was assessed using the Global Moran's I statistic to determine if the pattern of unimproved sanitation was clustered, dispersed, or random. A statistically significant and positive Moran's I value indicates spatial clustering of similar values(Novotný & Mamo, 2022).

Second, Getis-Ord Gi* hotspot analysis was performed to identify the locations of statistically significant hot spots (areas with high prevalence of unimproved sanitation) and cold spots (areas

with low prevalence) (Novotný & Mamo, 2022). Third, ordinary Kriging spatial interpolation was used to predict the prevalence of unimproved sanitation in unsampled areas based on the values from sampled clusters, creating a continuous risk map for the entire country. Finally, a purely spatial scan statistic using a Bernoulli model was employed in SaTScan software to detect significant spatial clusters of unimproved sanitation. Households with unimproved facilities were treated as cases, and those with improved facilities as controls, to identify geographic areas with a higher-than-expected prevalence.

2.7 Ethical Statement

This study is based on a secondary analysis of publicly available and anonymized data from the Somalia Integrated Household Budget Survey (SIHBS 2022). The original survey protocol was reviewed and approved by the relevant institutional review board in Somalia. The implementing agency, the Somalia National Bureau of Statistics (SNBS), obtained informed consent from all participants before data collection. Permission to use the dataset for this study was obtained through the official data archive channels.

3. Results

3.1 Univariate Analysis

The univariate analysis, detailed in **Table *1***, reveals that both individual and community-level characteristics are significantly associated with the use of unimproved sanitation facilities in Somalia. All variables presented demonstrated a statistically significant relationship with the outcome ($p < 0.001$ for most, with minor exceptions), underscoring the multifaceted nature of sanitation access.

Overall, 36.87% of Somali households rely on unimproved sanitation. This prevalence is situated within a range of recent estimates, being higher than the 22.3% reported in an analysis of the 2020 Somalia Health and Demographic Survey (SHDS) data (Ahmed & Ali, 2024) but lower than the 59.7% from the same 2020 survey data reported by another study (Azanaw et al., 2023). These discrepancies may reflect differences in variable definitions, weighting methodologies, or the dynamic nature of the country's humanitarian landscape between 2020 and the 2022 SIHBS survey period.

At the individual level, socioeconomic indicators emerged as powerful determinants. Education was a critical factor, with households headed by an individual with no formal schooling being significantly more likely to use unimproved facilities (44.02%) compared to those with some education (30.12%). This finding aligns with extensive research across sub-Saharan Africa, which consistently shows that education enhances awareness of hygiene-related health risks

and often correlates with higher economic status, enabling investment in better facilities (Azanaw et al., 2023; Belay & Andualem, 2022). Similarly, proxies for wealth, such as access to electricity and internet use, were strongly correlated with improved sanitation. For instance, nearly 60% of households without electricity used unimproved facilities, compared to just 23.12% of those with access. This stark divide highlights that unimproved sanitation is deeply embedded within a broader context of poverty and lack of access to modern infrastructure.

Vulnerability was another key theme. The data starkly illustrates the plight of Internally Displaced Persons (IDPs), with 64.27% of IDP households using unimproved facilities, compared to 35.64% of non-IDP households. This corroborates findings from humanitarian reports and studies on Somalia, which document the dire sanitation conditions in displacement camps where basic services are often overwhelmed or absent (Ahmed & Ali, 2024). Furthermore, food-insecure households reported significantly higher rates of unimproved sanitation (46.76%) than food-secure households (25.17%), underscoring a syndemic relationship where different forms of deprivation—lack of food, shelter, and sanitation—cluster within the same vulnerable populations. Housing type also reflected this, with over 61% of those in temporary or informal housing lacking improved sanitation, a finding consistent with studies on informal settlements globally(Spears et al., 2013).

At the community level, the type of residence was the most dramatic determinant. Nomadic populations face the most extreme challenges, with an overwhelming 83.28% using unimproved sanitation. This is consistent with studies on pastoralist communities, whose mobile lifestyle makes investment in permanent sanitation infrastructure impractical, often leading to a high prevalence of open defecation (Demoze et al., 2025). A significant rural-urban divide was also evident, with 51.10% of rural households using unimproved facilities compared to 23.88% of urban households. This rural disparity is a well-documented phenomenon across sub-Saharan Africa, driven by lower investment, weaker governance, and less access to markets for sanitation products and services(Hopewell & Graham, 2014; Pullan et al., 2014).

Finally, the analysis revealed profound regional inequalities. The prevalence of unimproved sanitation ranged from an alarming 90.65% in Hiraan and 80.39% in Bay to as low as 3.70% in Lower Shabelle and 5.37% in the capital region of Banadir. These geographical disparities are likely a direct reflection of varying levels of stability, governance, conflict, and humanitarian presence across Somalia's federal member states. Regions like Hiraan and Bay have been epicenters of conflict and climate-related shocks, severely hampering infrastructure development, whereas more stable and urbanized regions like Banadir have benefited from greater investment and access to services. This wide variation underscores that a single national figure for sanitation access masks critical local realities and highlights the necessity for geographically-targeted interventions to address the diverse challenges across Somalia.

Table 1 Descriptive Statistics of Unimproved Sanitation Facilities in Somalia

Variable<br>*Individual-level variables*	Categories	Sanitation Facilities		Chi2	P-value
		Unimproved (%)	Improved (%)		
**Sanitation Facility**		36.87	63.13		
**Age of household head**	<20 years	32.74	67.26	47.3653	0.001
	20-29 years	34.71	65.29		
	30-39 years	38.97	61.03		
	40-49 years	40.45	59.55		
	50-59 years	41.59	58.41		
	60+ years	37.64	62.36		
**Sex of household head**	Male	38.58	61.42	12.7784	0.001
	Female	35.42	64.58		
**Ever Attended school**	Yes	30.12	69.88	247.5964	0.001
	No	44.02	55.98		
**Marital Status**	Married	42.66	57.34	153.5151	0.001
	Divorced	31.27	68.73		
	Never married	31.51	68.49		
	Widowed	33.58	66.42		
**Housing type category**	Temporary/Informal/Basic	61.32	38.68	1300	0.001
	Permanent/Formal	26.79	73.21		
**Lighting-source**	Traditional/Other	58.86	41.14	1200	0.001
	Modern/Improved	26.15	73.85		
**Household Ownership**	Yes	36.53	63.47	4.7038	0.030
	No,non-household member	39.56	60.44		
**IDP**	Yes	64.27	35.73	173.5672	0.001
	No	35.64	64.36		
**Use Internet**	Yes	21.62	78.38	691.7784	0.001
	No	45.71	54.29		
**Electricity Access**	Yes	23.12	76.88	1600	0.001
	No	59.58	40.42		
**Labour_status**	Yes	39.04	60.96	3.6606	0.056
	No	36.54	63.46		
**Food security**	Food Secure	25.17	74.83	593.9134	0.001
	Food Insecure	46.76	53.24		
Community Level Variables					
**Type of Residence**	Rural	51.10	48.90	1800	0.001
	Urban	23.88	76.12		
	Nomadic	83.28	16.72		
**Region**	Awdal	44.13	55.87	2600	0.001
	Bakool	42.61	57.39		
	Banadir	5.37	94.63		
	Bari	11.38	88.62		
	Bay	80.39	19.61		
	Galgaduud	36.57	63.43		
	Gedo	21.27	78.73		
	Hiraan	90.65	9.35		
	Lower Juba	53.09	46.91		
	Lower Shabelle	3.70	96.30		
	Waqooyi Galbeed	41.67	58.33		
	Middle Shabelle	18.68	81.32		
	Mudug	29.01	70.99		

	Nugaal	52.02	47.98
	Sanaag	50.54	49.46
	Sool	27.62	72.38
	Togdheer	27.78	72.22

3.2 Multilevel Logistic Regression

The multilevel logistic regression analysis was conducted in a stepwise manner to disentangle the individual and community-level determinants of unimproved sanitation in Somalia, with the results presented in Table 2. This approach allows for a comparison between models to determine the most reliable and comprehensive explanation for the observed sanitation disparities.The process began with an empty model (Model 0), which contained no predictor variables. This model revealed a very high intraclass correlation coefficient (ICC) of 0.94, indicating that 94% of the total variance in the use of unimproved sanitation can be attributed to differences between communities (enumeration areas). This finding strongly justified the use of a multilevel model, as it confirms that household sanitation status is heavily dependent on community-level context.

Model 1 focused exclusively on individual-level factors. This model identified households living in permanent/formal housing (AOR: 3.67; 95% CI: 2.97, 4.53) and those with internally displaced person (IDP) status (AOR: 3.37; 95% CI: 2.16, 5.23) as having significantly higher odds of using unimproved sanitation. This highlights the severe vulnerability of displaced populations and suggests a paradox where "permanent" housing in camp-like settings may lack basic services. Conversely, factors often associated with poverty, such as lacking electricity (AOR: 0.39) or internet access (AOR: 0.68), were paradoxically associated with lower odds, likely because these households are concentrated in areas with different sanitation challenges (e.g., rural open defecation) not fully captured by this model's scope. While insightful, Model 1 only tells part of the story by ignoring the broader community context.

Model 3, the full and most reliable model, integrated both individual and community-level variables. Its superiority is statistically confirmed by its significantly lower AIC (7356.637) and BIC (7637.396) values compared to all other models (as shown in Table 3), indicating the best model fit. By including both sets of factors, this model provides the most nuanced and accurate understanding of the issue.

Comparing **Model 1** and **Model 3** reveals both similarities and crucial differences in interpretation. The strong effects of individual-level factors like permanent/formal housing (AOR: 3.42) and IDP status (AOR: 3.18) remained highly significant and of a similar magnitude in Model 3. This similarity demonstrates that these household-level vulnerabilities are robust predictors of unimproved sanitation, even after accounting for community-level differences. Their influence is direct and powerful regardless of geographic location.

However, the inclusion of community-level variables in **Model 3** fundamentally changes the overall picture. The model shows that community context is an overwhelmingly powerful driver that reshapes the risk landscape. For instance, urban residence was associated with dramatically higher odds of unimproved sanitation (AOR: 7.99) compared to rural areas, while nomadic populations had exceptionally lower odds (AOR: 0.04), likely reflecting a high prevalence of open

defecation not classified as a "facility." Most strikingly, regional disparities were extreme, with residents in Lower Shabelle having an astonishingly high odds (AOR: 175.42) compared to the reference region of Awdal, while those in Hiraan and Bay had significantly lower odds.

In conclusion, **Model 3 is the most reliable** because it accounts for the hierarchical nature of the data and demonstrates that while individual household characteristics are important, they operate within a much larger context of community and regional factors. The difference between Model 1 and Model 3 is that the latter clarifies that an individual's risk is profoundly modified by where they live. Therefore, a comprehensive understanding requires considering both the specific circumstances of a household (like displacement) and the overarching structural realities of their community (such as urban infrastructure deficits or regional instability).

Table 2. Multilevel Analysis Unimproved Sanitation Facilities in Somalia

Variable	Categories	Model 0: Empty Model (AOR [95% CL])	Model 1: Individual Factors (AOR [95% CL])	Model 2: Community Factors (AOR [95% CL])	Model 3: Full Model (AOR [95% CL])
**Age of household head**	20-29 years		0.83 [0.67, 1.04]		0.84 [0.67,1.05]
	30-39 years		0.82 [0.64, 1.06]		0.81 [0.63,1.04]
	40-49 years		0.94 [0.69, 1.27]		0.92 [0.68,1.25]
	50-59 years		0.83 [0.60, 1.16]		0.81 [0.58,1.13]
	60+ years		0.89 [0.65, 1.20]		0.86 [0.63, 1.16]
**Sex of household head**	Female		1.14 [0.99, 1.31]		1.13 [0.98, 1.30]
**Ever Attended school**	No		1.03 [0.87, 1.21]		1.02 [0.87, 1.20]
**Marital Status**	Divorced		0.90 [0.66, 1.23]		0.88 [0.64, 1.21]
	Never married		0.88 [0.70, 1.09]		0.85 [0.68, 1.06]
	Widowed		1.08 [0.82, 1.43]		1.06 [0.80, 1.40]
**Housing type category**	Permanent/Formal		3.67 [2.97, 4.53]		3.42 [2.76, 4.23]
**Lighting-source category**	Modern/Improved		0.88 [0.65, 1.19]		0.84 [0.62, 1.13]
**Household Ownership**	No, non-household member		0.69 [0.55, 0.86]		0.69 [0.55, 0.87]
**IDP**	No		3.37 [2.16, 5.23]		3.18 [2.06, 4.92]
**Use Internet**	No		0.68 [0.57, 0.80]		0.71 [0.60, 0.84]
**Electricity Access**	No		0.39 [0.28, 0.54]		0.44 [0.32, 0.60]
**Labour_status**	No		1.04 [0.85, 1.28]		1.04 [0.85, 1.28]
**Food security**	Food Insecure		0.63 [0.54, 0.75]		0.66 [0.56, 0.78]
**Type of Residence**	Urban			14.46 [7.70, 27.15]	7.99 [4.35,14.69]
	Nomadic			0.01 [0.004, 0.035]	0.04 [0.01,0.11]
**Region**	Bakool			0.66 [0.14, 3.05]	1.80 [0.41, 7.83]
	Banadir			23.18 [5.05,106.45]	17.12[3.88,75.61]
	Bari			12.37 [2.67, 57.34]	11.64[2.6, 52.6]
	Bay			0.04 [0.01, 0.2]	0.08[0.02, 0.39]
	Galgaduud			1.73 [0.40, 7.44]	2.39 [0.58, 9.82]
	Gedo			6.01 [1.31, 27.46]	11.98 [2.68,53.5]
	Hiraan			0.0009 [0.0001, 0.0051]	0.003[0.001,0.02]
	Lower Juba			0.29 [0.06,1.46]	0.37 [0.08, 1.70]
	Lower Shabelle			213.19 [27.92,1627.65]	175 [24.4,1259.9]
	Waqooyi Galbeed			1.34 [0.35, 5.07]	1.26 [0.35, 4.58]
	Middle Shabelle			7.09 [1.63, 30.78]	12.77 [3.02, 54]
	Mudug			2.64 [0.57, 12.29]	2.94 [0.66, 13.07]
	Nugaal			0.15 [0.03, 0.73]	0.15 [0.03, 0.67]

	Sanaag	0.39 [0.08, 1.80]	0.36 [0.08, 1.57]
	Sool	4.96 [1.03, 23.78]	4.75 [1.05, 21.41]
	Togdheer	14.77 [3.21, 67.87]	17.00 [3.80, 76.1]

3.3 Logistic Regression Models comparison

The model's comparison in **Table *3*** provides a clear progression in model fit and explanation of variance regarding unimproved sanitation facilities in Somalia. The initial Empty Model (Model 0), with a Log-likelihood of -3978.9811, an AIC of 7961.962, and a BIC of 7976.739, reveals a substantial intraclass correlation coefficient (ICC) of 0.9396. This high ICC indicates that approximately 94% of the total variance in unimproved sanitation is attributable to differences between clusters (enumeration areas), underscoring the necessity of a multilevel approach. As individual-level variables are introduced in Model 1, the Log-likelihood improves to -3797.8329, while the AIC (7635.666) and BIC (7783.434) decrease, signifying a better model fit. The ICC also drops to 0.8792, suggesting that individual household characteristics explain some of the variance in sanitation status. Model 2, incorporating only community-level variables, shows an even greater improvement, with a Log-likelihood of -3784.4915 and further reductions in AIC (7608.983) and BIC (7756.751). The ICC in this model is 0.7786, indicating that community-level factors account for a significant portion of the variability in unimproved sanitation. Finally, Model 3, the Full Model, which includes both individual and community-level variables, demonstrates the best fit among all models. It achieves the highest Log-likelihood of -3640.3183 and the lowest AIC (7356.637) and BIC (7637.396). The ICC in the Full Model is 0.7418, the lowest of all models, implying that the combined individual and community-level factors substantially reduce the unexplained variance between clusters. The consistent decrease in AIC, BIC, and ICC values across the models, culminating in the Full Model, confirms that a multilevel approach integrating both individual and community-level determinants is essential for comprehensively understanding the factors influencing unimproved sanitation facilities in Somalia.

Table 3 Models comparison

Model	N	Log-likelihood	AIC	BIC	ICC
**Model 0 (Empty)**	11,949	-3978.9811	7961.962	7976.739	0.9396
**Model 1 (Individual)**	11,949	-3797.8329	7635.666	7783.434	0.8792
**Model 2 (Community)**	11,949	-3784.4915	7608.983	7756.751	0.7786

**Model 3 (Full)**	11,949	-3640.3183	7356.637	7637.396	0.7418

3.4 Spatial Analysis Result

3.4.1 Spatial distribution

The spatial distribution of unimproved sanitation facilities in Somalia, depicted in *Figure 1*, reveals profound regional inequalities that mirror the country's complex landscape of security, governance, and development. The analysis identifies critical hotspots in the **Hiraan (90.65%)** and **Bay (80.39%)** regions, where access to basic sanitation is almost non-existent for the vast majority of the population. This dire situation is a direct consequence of these regions being epicenters of protracted conflict, severe climate shocks, and political instability. The persistent insecurity destroys existing infrastructure, prevents new development projects from being implemented, and severely limits the operational capacity of humanitarian and development organizations. Furthermore, these regions host large populations of internally displaced persons (IDPs), who are often congregated in camps where sanitation facilities are overwhelmed or entirely absent, thus driving up the regional prevalence of unimproved sanitation.

In stark contrast, the regions of **Banadir (5.37%)** and **Lower Shabelle (3.70%)** emerge as significant cold spots, indicating far better access to improved sanitation. The success in **Banadir**, which contains the capital city Mogadishu, can be attributed to its status as the seat of government and the primary hub for international partners, NGOs, and private investment. This concentration of resources has led to greater investment in urban infrastructure and public services compared to other parts of the country. Similarly, the low prevalence in **Lower Shabelle** is likely influenced by its proximity to the capital and its role as a key agricultural region, which supports more settled communities and has historically seen greater development focus.

Between these extremes lie regions with moderate to high challenges, such as Lower Juba, Nugaal, and Sanaag, which all report prevalence rates above 50%. Ultimately, the map does not merely show sanitation coverage; it provides a clear visual proxy for underlying disparities in stability, governance, economic development, and humanitarian presence across Somalia, underscoring the necessity of geographically-targeted interventions to address these deep-rooted inequalities.

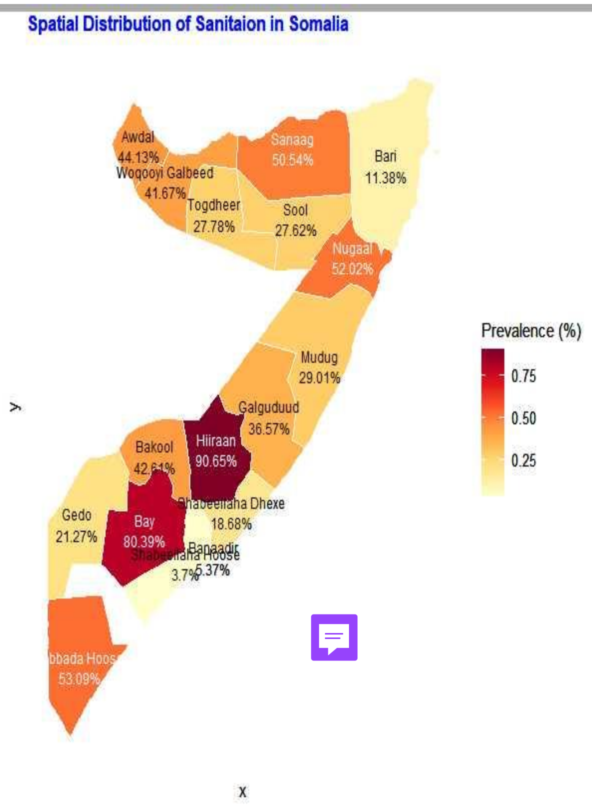

Figure 1 Spatial Distribution of Unimproved Sanitation Facilities

3.4.2 Spatial Autocorrelation-Global Moran's I

Figure 2, the Global Moran's I normal distribution curve, indicates that the spatial distribution of unimproved sanitation facilities in Somalia is not statistically clustered or dispersed. With a Moran's Index of -0.19894, a z-score of -0.75544, and a high p-value of 0.77501, the data suggests a random spatial pattern, meaning there's no significant global clustering of high or low prevalence areas.

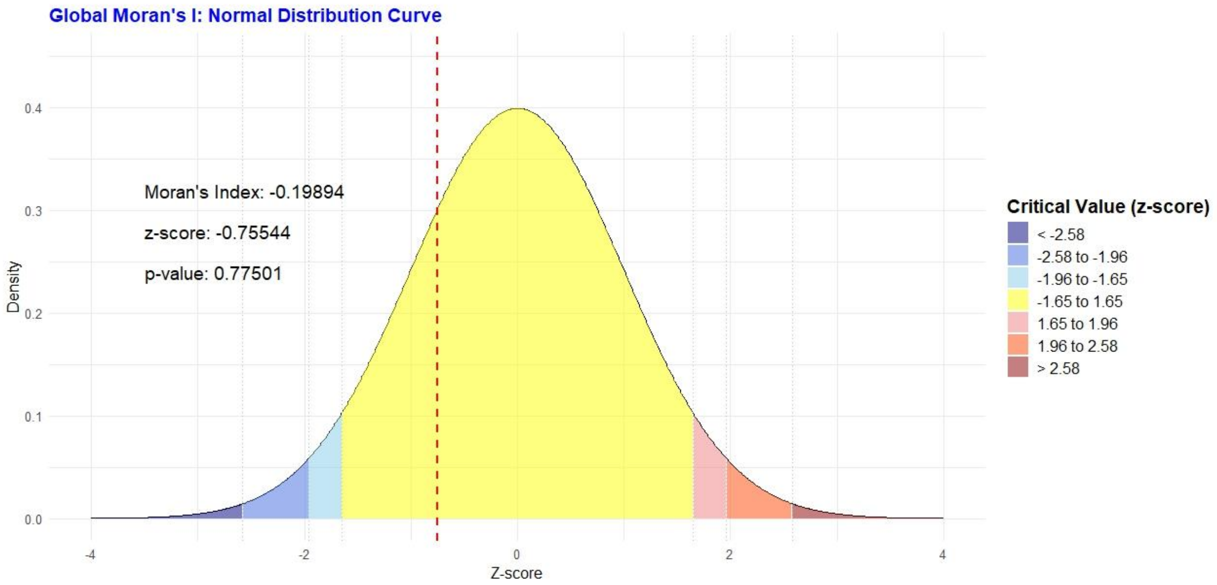

Figure 2 Global Moran's I for spatial Autocorrelation of unimproved sanitation facilities

3.4.3 Spatial autocorrelation-local Moran's I

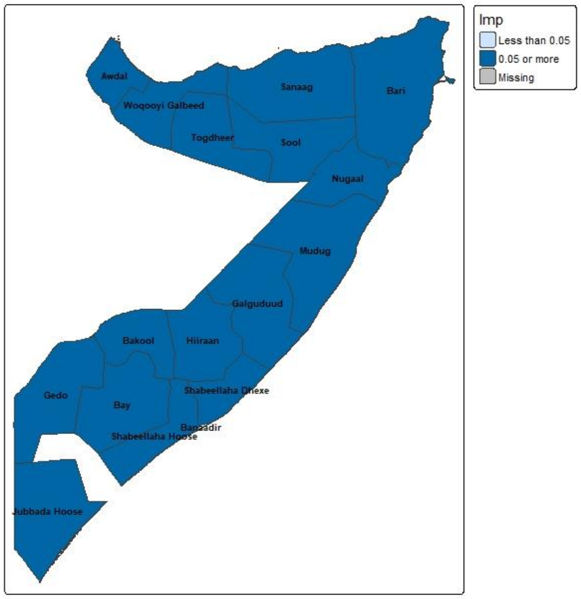

Figure 4 and Figure 4,composed of two sets of maps, illustrates the localized patterns of unimproved sanitation facilities in Somalia using Local Moran's I and corresponding p-values. The maps on the left side (grey with blue patches) identify specific areas where local spatial clustering is statistically significant, meaning the prevalence of unimproved sanitation in those blue regions is not random compared to their neighbors. The maps on

the right, which are almost entirely dark blue, strongly indicate that for nearly all regions across Somalia, the observed local spatial patterns of unimproved sanitation are statistically significant (p-value less than 0.05). This means that, despite a lack of overall global clustering, there are distinct and statistically meaningful local concentrations either "hotspots" of high unimproved sanitation or "coldspots" of low unimproved sanitation across the vast majority of the country's administrative divisions.

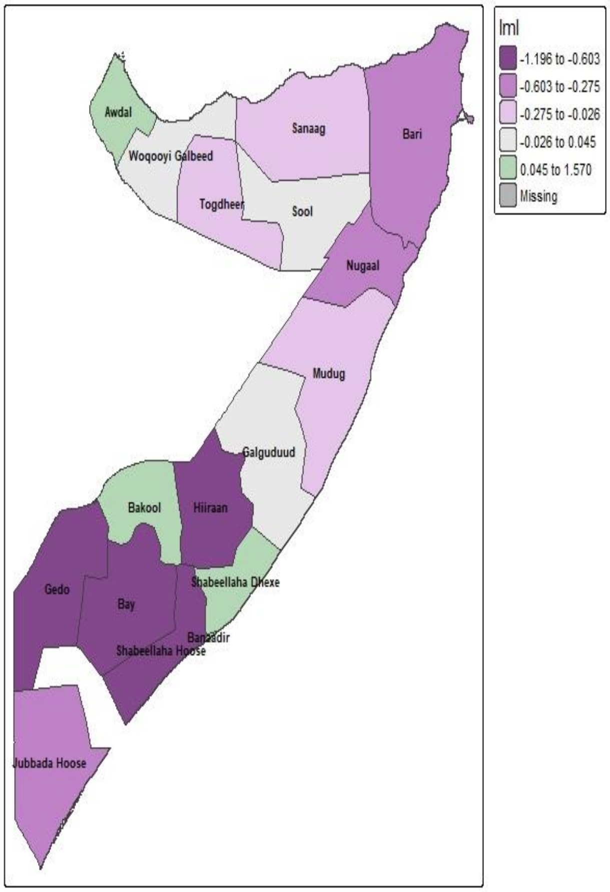

Figure 3 Local Moran's I of unimproved sanitation

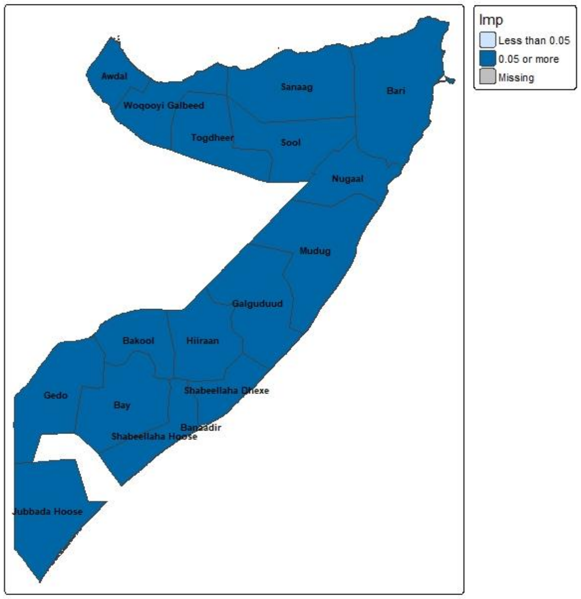

Figure 4 Local Moran's I and its p-value for significance

3.4.4 Hot spot and cold spot analysis

Figure *5*, displaying the Getis-Ord Gi* results, maps the spatial distribution of statistically significant hot spots and cold spots of unimproved sanitation facilities across Somalia. The color gradient in the legend indicates the range of Gi* values, which identify areas with high values (hot spots) or low values (cold spots) clustered together. Darker green shades (0.615 to 1.603) represent statistically significant hot spots, indicating regions where high prevalence of unimproved sanitation facilities is clustered. We can observe these hot spots prominently in areas like Hiraan, Bay, Galgaduud, Gedo, Mudug, and parts of the northern regions such as Nugaal. Conversely, the lighter purple shades (-1.847 to -0.979) represent statistically significant cold spots, signifying clusters of low prevalence of unimproved sanitation facilities. These cold spots appear in regions like Awdal, Juba Hoose, and parts of the eastern coast (Bari). Intermediate shades of purple and green suggest areas with less extreme clustering or values closer to the mean. This Getis-Ord Gi* analysis visually corroborates and further refines the understanding of spatial disparities, clearly pinpointing specific regions that are hotbeds of unimproved sanitation and those with better access, thereby providing critical insights for geographically targeted public health interventions.

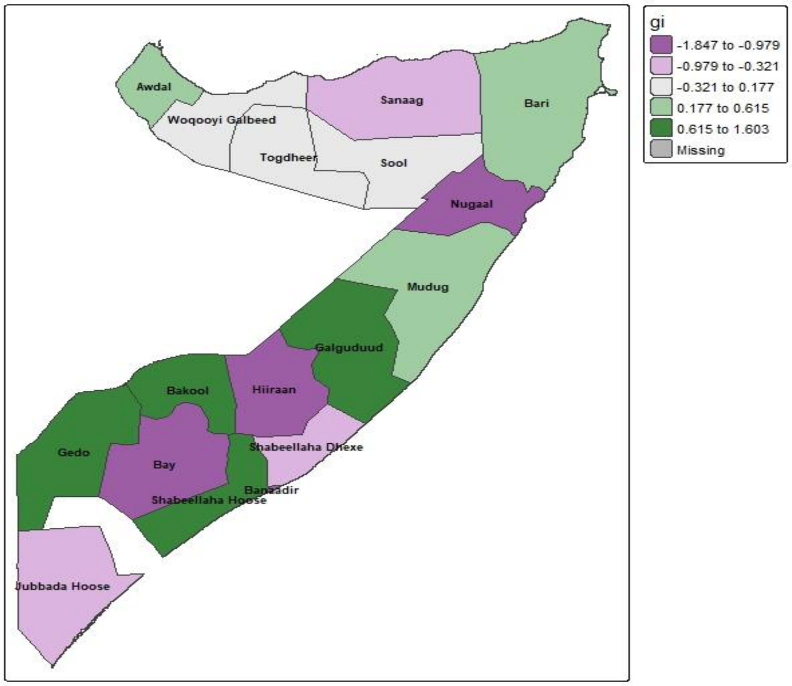

Figure 5 Hot and cold spots region using Getis-Ord G*

4. Discussion

This study's multilevel and spatial analysis provides a granular understanding of the complex determinants driving the use of unimproved sanitation in Somalia, with findings that both corroborate and challenge existing literature. The overall prevalence of over one-third of households using unimproved facilities highlights a persistent public health crisis. This figure is situated within a range of estimates for Somalia, including a 2024 study using 2020 data that reported 22.3% of households using unimproved facilities and an earlier survey indicating a higher rate of 59.7% (Saleem et al., 2019; Unicef, 2018). These variations likely reflect differences in survey methodologies and the dynamic nature of displacement and infrastructure challenges in the country.

A key finding is the stark disparity across residence types, with nomadic populations exhibiting an overwhelming 83.28% prevalence of unimproved sanitation. This is consistent with the understanding that nomadic lifestyles present significant challenges for fixed sanitation infrastructure, often leading to a reliance on open defecation (Demoze et al., 2025; Pullan et al., 2014). The significantly lower odds of unimproved sanitation among nomadic groups in the multilevel model (AOR: 0.04) is a statistical reflection of open defecation not being classified as a

facility, rather than indicating better access. In contrast, the high odds for urban residents (AOR: 7.99) compared to their rural counterparts' points to a crisis of rapid, unplanned urbanization outstripping the provision of basic services, a phenomenon observed in other rapidly growing urban centers in sub-Saharan Africa. A recent study in Ethiopia, for example, found that while urban areas overall had better access, certain urban populations were highly vulnerable (J. Brown et al., 2013; Yusuf et al., 2023c).

The multilevel analysis revealed several nuanced and seemingly paradoxical associations. The finding that households in permanent/formal housing had significantly higher odds of using unimproved sanitation (AOR: 3.42) is counterintuitive but may be explained by the context of protracted displacement. Many internally displaced persons (IDPs) live in camps where the structures are considered "permanent," yet they lack adequate sanitation services (Dahir et al., 2024; Spears et al., 2013). This is strongly supported by the finding that IDP status itself dramatically increases the odds of using unimproved sanitation by more than threefold (AOR: 3.18), confirming the extreme vulnerability of this population group, a fact well-documented in humanitarian reports on Somalia (Dahir et al., 2024; ESCAP, 2016; Spears et al., 2013)[.

The spatial analysis was instrumental in moving beyond national averages to identify specific geographic inequities. The identification of Hiraan (90.65%) and Bay (80.39%) as critical hotspots aligns with the known impacts of conflict, climate shocks, and instability on these regions, which severely impede infrastructure development. Conversely, the significantly better outcomes in the more urbanized regions of Banadir (5.37%) and Lower Shabelle (3.70%) underscore the importance of governance and the presence of humanitarian and development actors. The Getis-Ord Gi* analysis, by confirming these statistically significant hot and cold spots, provides a clear evidence base for geographically targeted interventions. While the Global Moran's I indicated no overall global clustering, the significant local clustering reinforces the idea that the drivers of poor sanitation are highly localized. This aligns with broader research across sub-Saharan Africa, which increasingly calls for context-specific, decentralized approaches to address sanitation challenges effectively (Azanaw et al., 2023; Hopewell & Graham, 2014; Osumanu et al., 2019).

5. Conclusion

This study reveals that the state of sanitation in Somalia is a crisis of deep and geographically entrenched inequalities, driven by a complex interplay of socioeconomic vulnerability, displacement, and lifestyle. The overall prevalence of over one-third of households using unimproved sanitation masks profound disparities, with the burden falling disproportionately on

nomadic populations (83.28%), rural communities, and the internally displaced. The multilevel analysis moved beyond simple prevalence to uncover critical, and at times paradoxical, determinants. Counterintuitively, households in permanent/formal housing and those with IDP status faced significantly higher odds of using unimproved facilities. This highlights a critical reality in Somalia: many IDPs reside in camp-like settings with "permanent" structures that lack basic services, underscoring that housing without integrated sanitation infrastructure exacerbates vulnerability.

Furthermore, the findings challenge a simplistic urban-rural dichotomy. While urban areas have better sanitation coverage overall, the multilevel model showed urban residence was paradoxically associated with higher odds of unimproved sanitation compared to rural areas. This points to a crisis of rapid, unplanned urbanization where the growth of informal settlements is far outstripping the provision of essential services. Similarly, while nomadic populations have the highest prevalence of unimproved sanitation, their exceptionally low odds in the model reflect a statistical nuance where the widespread practice of open defecation is not captured as a "facility," rather than indicating better access.

The spatial analysis provides a clear roadmap for intervention, confirming that the drivers of poor sanitation are highly localized. While no single national pattern of clustering was found, significant local hot spots and cold spots were identified. The regions of Hiraan and Bay emerge as critical hot spots, where protracted conflict and climate shocks have decimated infrastructure and created a public health emergency. In stark contrast, the cold spots in the more stable and urbanized regions of Banadir and Lower Shabelle underscore the powerful role that governance, security, and humanitarian presence play in enabling access to services.

Ultimately, tackling Somalia's sanitation crisis demands a multi-pronged and evidence-informed approach. Interventions must be geographically targeted, prioritizing the identified hotspots with urgent investment in basic infrastructure. Policies must be tailored to the distinct needs of urban, rural, and nomadic populations, with a special focus on the highly vulnerable IDP communities by ensuring housing projects are always accompanied by essential water and sanitation services. Addressing these deep-rooted inequalities is not merely an infrastructure challenge; it is a critical imperative for advancing public health, upholding human dignity, especially for women and girls, and achieving sustainable development for all in Somalia.

Reference

Ahmed, H. A., & Ali, D. A. (2024). Prevalence and determinants of household access to improved latrine utilization in Somalia: health demographic survey (SHDS) 2020. *Environmental Health Insights*, *18*, 11786302241284148.

Azanaw, J., Abera, E., Malede, A., & Endalew, M. (2023). A multilevel analysis of improved drinking water sources and sanitation facilities in Ethiopia: Using 2019 Ethiopia mini demographic and health survey. *Frontiers in Public Health*, *11*, 1063052.

Belay, D. G., & Andualem, Z. (2022). Limited access to improved drinking water, unimproved drinking water, and toilet facilities among households in Ethiopia: Spatial and mixed effect analysis. *PLoS ONE*, *17*(4 April), 1–20. https://doi.org/10.1371/journal.pone.0266555

Belay, D. G., Asratie, M. H., Aragaw, F. M., Tsega, N. T., Endalew, M., & Gashaw, M. (2022). Open defecation practice and its determinants among households in sub-Saharan Africa: pooled prevalence and multilevel analysis of 33 sub-Saharan Africa countries demographic and health survey. *Tropical Medicine and Health*, *50*(1), 28.

Brown, C., Neves-Silva, P., & Heller, L. (2016). The human right to water and sanitation: a new perspective for public policies. *Ciencia & Saude Coletiva*, *21*, 661–670.

Brown, J., Cairncross, S., & Ensink, J. H. J. (2013). Water, sanitation, hygiene and enteric infections in children. *Archives of Disease in Childhood*, *98*(8), 629–634.

Dahir, H. G., Ahmed, A. T., & Abdilahi, M. A. (2024). *Burden and Determinants of Child Mortality in Somalia analysis of SDHS 2020*.

Demoze, L., Tigabie, M., Worede, E. A., Brhan, H., Gizaw, Z., Tesfaye, A. H., & Yitageasu, G. (2025). Determinants and geographic distribution of unimproved sanitation facilities in sub-Saharan Africa, spatial and multilevel analysis using demographic and health survey (DHS) data. *BMC Public Health*, *25*(1), 2848.

Demsash, A. W., Tegegne, M. D., Wubante, S. M., Walle, A. D., Donacho, D. O., Senishaw, A. F., Emanu, M. D., & Melaku, M. S. (2023). Spatial and multilevel analysis of sanitation service access and related factors among households in Ethiopia: using 2019 Ethiopian national dataset. *PLOS Global Public Health*, *3*(4), e0001752.

Desye, B., Geto, A. K., Daba, C., Bezie, A. E., Reda, S., Sisay, T., Getachew, M., & Berhanu, L. (2025). Spatial and multilevel analysis of determinant factors for safely managed sanitation services in Ethiopia. *Scientific Reports*, *15*(1), 31027.

ESCAP, U. N. (2016). *Sustainable Development Goal 6: ensure availability and sustainable management of water and sanitation for all*.

Heijnen, M., Cumming, O., Peletz, R., Chan, G. K.-S., Brown, J., Baker, K., & Clasen, T. (2014). Shared sanitation versus individual household latrines: a systematic review of health outcomes. *PloS One*, *9*(4), e93300.

Hopewell, M. R., & Graham, J. P. (2014). Trends in access to water supply and sanitation in 31 major sub-Saharan African cities: an analysis of DHS data from 2000 to 2012. *BMC Public Health*, *14*(1), 208.

Novotný, J., & Mamo, B. G. (2022). Household - level sanitation in Ethiopia and its influencing factors : a systematic review. *BMC Public Health*, 1–16. https://doi.org/10.1186/s12889-022-13822-5

Organization, W. H. (2022). *Country cooperation strategy for WHO and Somalia 2021–2025*.

Osumanu, I. K., Kosoe, E. A., & Ategeeng, F. (2019). Determinants of open defecation in the Wa municipality of Ghana: empirical findings highlighting sociocultural and economic dynamics among households. *Journal of Environmental and Public Health*, *2019*(1), 3075840.

Pullan, R. L., Freeman, M. C., Gething, P. W., & Brooker, S. J. (2014). Geographical inequalities in use of improved drinking water supply and sanitation across sub-Saharan Africa: mapping and spatial analysis of cross-sectional survey data. *PLoS Medicine*, *11*(4), e1001626.

Saleem, M., Burdett, T., & Heaslip, V. (2019). Health and social impacts of open defecation on women: a systematic review. *BMC Public Health*, *19*, 1–12.

Spears, D., Ghosh, A., & Cumming, O. (2013). Open defecation and childhood stunting in India: an ecological analysis of new data from 112 districts. *PloS One*, *8*(9), e73784.

Unicef. (2018). *Drinking water, sanitation and hygiene in schools: global baseline report 2018*.

Yusuf, A. M., Pasukphun, N., & Suma, Y. (2023a). Factors associated with sanitation practices among household-level in Mogadishu, Somalia. *International Journal of Public Health Asia Pacific*, *August*. https://doi.org/10.62992/ijphap.v2i2.24

Yusuf, A. M., Pasukphun, N., & Suma, Y. (2023b). Factors associated with sanitation practices among household-level in Mogadishu, Somalia. *International Journal of Public Health Asia Pacific*.

Yusuf, A. M., Pasukphun, N., & Suma, Y. (2023c). Factors associated with sanitation practices among household-level in Mogadishu, Somalia. *International Journal of Public Health Asia Pacific*, *2*(02), 1–12. https://doi.org/10.62992/ijphap.v2i2.24

Spatial Distribution and Determinants of Unimproved Sanitation Facilities Among Households in Somalia: Using Somalia Integrated Household Budget Survey (SIHBS 2022)

Omar Muhumed Maidhane[1,3], Omran Salih[1,4,*], Abdisalam Hassan Muse[1,2], Abdirahman Omer Osman[1], Muse H Abdi[1], Mahdi Hashi Hassan[1], Nur Mohamud Ali[1], Shacban Abdilahi Elmi[1]

[1]School of Postgraduate Studies and Research, Amoud University, Borama 25263, Somalia

[2]Research and Innovation Center, Amoud University, Borama 25263, Somalia

[3]Research and Innovation Center, Galkayo University, Galmudug, Somalia.

[4]Institute of System Science, Durban University of Technology, Durban, South Africa

[*]Corresponding Author: *Omar Muhumed Maidhane*

Abstract

Background: Access to adequate sanitation remains a critical public health challenge in Somalia, where a large portion of the population relies on unimproved facilities due to persistent conflict, climate shocks, and political instability. This reliance contributes to a high burden of waterborne diseases. This study aimed to assess the spatial distribution of unimproved sanitation and identify its individual and community-level determinants using recent national data to inform targeted interventions.

Methods: This study is a secondary analysis of the 2022 Somalia Integrated Household Budget Survey (SIHBS), which included 7,212 households. The primary outcome was the use of unimproved sanitation facilities, categorized according to the WHO/UNICEF Joint Monitoring Programme (JMP) definitions. We employed a multilevel logistic regression model to identify individual and community-level determinants associated with unimproved sanitation. To analyze the spatial patterns of unimproved sanitation, we used Global Moran's I for spatial autocorrelation and the Getis-Ord Gi* statistic for hotspot analysis.

Results: Overall, 36.87% of Somali households use unimproved sanitation facilities. There are significant disparities across residence types, with the highest prevalence among nomadic populations (83.28%), followed by rural (51.10%) and urban (23.88%) residents. The multilevel analysis revealed that households in permanent/formal housing (AOR: 3.42) and those with IDP status (AOR: 3.18) had significantly higher odds of using unimproved sanitation. At the community level, urban residence was paradoxically associated with higher odds of unimproved sanitation (AOR: 7.99) compared to rural areas, while nomadic populations had significantly lower odds (AOR: 0.04), likely reflecting a high prevalence of open defecation not captured as a "facility." Spatial analysis identified significant hotspots of unimproved sanitation in the Hiraan (90.65%) and Bay (80.39%) regions, and cold spots in Banadir (5.37%) and Lower Shabelle (3.70%).

Conclusion: The findings highlight deep inequalities in sanitation access across Somalia, driven by geographic location, socioeconomic status, and population group. The high prevalence of unimproved sanitation, especially among nomadic, rural, and displaced populations, calls for urgent, geographically-targeted interventions. A multi-pronged approach is necessary, focusing on the specific needs of different communities and addressing the underlying structural and individual-level drivers of poor sanitation to advance public health and sustainable development goals in the region.

Keywords: Unimproved sanitation; Spatial analysis; Multilevel modeling; Somalia; Public health disparities

1. Introduction

Access to adequate and equitable sanitation is a fundamental human right and a critical component of public health and sustainable development (Yusuf et al., 2023a). Globally, 3.5 billion people still lacked safely managed sanitation in 2022, with 419 million practicing open defecation (Saleem et al., 2019). The disease burden resulting from inadequate sanitation is immense, contributing to the transmission of diseases like cholera, typhoid, and intestinal worm infections, which disproportionately affect children under five (Demoze et al., 2025; Unicef, 2018). Poor sanitation is a significant factor in environmental degradation and is closely linked to childhood stunting and malnutrition (Demoze et al., 2025; Pullan et al., 2014). The Sustainable Development Goals (SDGs), particularly Target 6.2, aim to achieve universal access to sanitation for all and end open defecation by 2030, paying special attention to the needs of women, girls, and those in vulnerable situations (Yusuf et al., 2023b).

Sub-Saharan Africa faces the most significant challenges in sanitation coverage, with disparities between urban and rural areas and slow overall progress (J. Brown et al., 2013; Spears et al., 2013). Somalia, in particular, confronts acute sanitation challenges exacerbated by decades of conflict, political instability, climate-related disasters such as recurrent droughts, and large-scale displacement (Ahmed & Ali, 2024; Yusuf et al., 2023b). According to the WHO/UNICEF Joint Monitoring Programme (JMP), access to even basic sanitation facilities in Somalia is critically low, with a significant portion of the population, especially in rural and nomadic communities, resorting to unimproved facilities or open defecation (ESCAP, 2016; Hopewell & Graham, 2014). Recent data from the 2020 Somalia Demographic Health and Survey indicated that a substantial proportion (59.7%) of households utilized unimproved sanitation facilities (Azanaw et al., 2023). This situation poses severe risks to public health, dignity, and safety, particularly for women and girls who face heightened risks of harassment and violence when seeking private places to relieve themselves (Osumanu et al., 2019).

Despite the clear urgency, there is limited comprehensive research on the specific factors driving the prevalence of unimproved sanitation in Somalia. While several studies have highlighted the scale of the problem, a detailed analysis that combines both spatial and multilevel approaches to understand the geographic disparities and the interplay of individual and community-level determinants is lacking (Belay et al., 2022; Unicef, 2018). Such an analysis is crucial for designing evidence-based, targeted interventions that can address the root causes of poor sanitation. Therefore, this study aims to assess the spatial distribution and identify the determinants of unimproved sanitation facilities among households in Somalia, using recent data from the Somalia Integrated Household Budget Survey (SIHBS 2022).

This study provides a novel and in-depth analysis of sanitation challenges in Somalia by being one of the first to combine a multilevel modeling approach with advanced spatial analysis. While previous research has acknowledged the poor state of sanitation in the country, this study moves beyond descriptive statistics to simultaneously investigate both individual and community-level determinants of unimproved sanitation. The use of the recent and comprehensive 2022 Somalia Integrated Household Budget Survey (SIHBS) dataset allows for a more current and granular understanding of the issue. Furthermore, the application of geospatial techniques such as Getis-Ord Gi* hotspot analysis and spatial autocorrelation provides a clear visual and statistical identification of geographic disparities, pinpointing specific regions and communities that are most affected. This dual approach offers a more robust evidence base for policymakers and public health stakeholders, enabling the design of targeted, context-specific interventions that address both the socioeconomic drivers and the geographical clustering of poor sanitation in Somalia.

2. Material And Methods

2.1 Study Area

The study was conducted in Somalia, a country in the Horn of Africa with an estimated 2024 population of around 19 million and a low population density of approximately 32 people per square kilometer. The nation's economy is primarily agrarian, with the livestock sector accounting for a significant portion of the GDP and over half of export earnings. Despite recent economic growth, Somalia is classified as a least-developed country, and in 2022, over half of the population lived below the national poverty line. Decades of civil conflict and severe climate shocks have devastated public infrastructure, including water and sanitation systems, leading to widespread displacement and vulnerability among its diverse urban, rural, and nomadic communities(Yusuf et al., 2023b).

2.2 Study Design and Data Source

This study employed a secondary analysis of data from the 2022 Somalia Integrated Household Budget Survey (SIHBS), a nationally representative, community-based cross-sectional survey. The SIHBS was conducted by the Somalia National Bureau of Statistics (SNBS) and is the first comprehensive household budget survey in the country since 1985 (Demsash et al., 2023).

The survey used a two-stage stratified cluster sampling design, covering urban, rural, and nomadic populations. In the first stage, enumeration areas (EAs) were selected with probability proportional to their size, followed by the systematic selection of a fixed number of households from each EA in the second stage. The survey included a total sample of 7,212 households. This analysis utilized the final weighted sample to ensure national representativeness.

2.3 Data validation

To ensure the findings are representative of the national population and to account for the complex survey design, sampling weights provided in the SIHBS dataset were applied during all statistical analyses. This weighting procedure adjusts for the unequal probability of selection and non-response rates, ensuring reliable and valid estimates.

2.4 Study variables

The primary outcome variable was the status of the household sanitation facility, categorized as a binary variable: "unimproved" or "improved." This classification was based on the WHO/UNICEF JMP definitions (Organization, 2022). "Unimproved sanitation facilities" were defined as pit latrines without a slab or platform, hanging latrines, bucket latrines, and the practice of open defecation(C. Brown et al., 2016). *This was coded as '1'.* "Improved sanitation facilities" included flush or pour-flush toilets connected to a piped sewer system, septic tanks, or pit latrines; ventilated improved pit latrines; and pit latrines with a slab, which are not shared with other households. *This was coded as '0'.*

The independent variables were classified into individual-level and community-level factors. Individual-level variables included the sex of the household head (male or female), age of the household head (categorized), educational attainment (no education, primary, secondary, or higher), household size, and the household wealth index, which was categorized into five quintiles: poorest, poorer, middle, richer, and richest. Community-level variables comprised the place of residence (urban, rural, or nomadic), region or federal member state, and community

media exposure, defined as the proportion of households within the enumeration area (EA) that had access to radio or television.

2.6 Data Analysis

2.6.1 Univariant and Multilevel Analysis

Data cleaning, coding, and analysis were performed using STATA version 17. Descriptive statistics, including frequencies and percentages, were used to summarize the characteristics of the study population and the prevalence of unimproved sanitation. Due to the hierarchical nature of the survey data, where households (level 1) are nested within enumeration areas or clusters (level 2), a multilevel logistic regression model was employed to identify the determinants of unimproved sanitation (Desye et al., 2025). This approach accounts for the clustering effect and avoids the underestimation of standard errors that can occur with standard logistic regression (Demoze et al., 2025; Heijnen et al., 2014).

Four consecutive models were fitted: a null model (Model 0) with no explanatory variables to assess the variance in unimproved sanitation between clusters; Model I included only individual-level variables; Model II included only community-level variables; and Model III, the final model, included both individual and community-level variables simultaneously. Measures of variation (random effects) such as the Intraclass Correlation Coefficient (ICC), Median Odds Ratio (MOR), and Proportional Change in Variance (PCV) were calculated to quantify the extent of clustering. Model fit was assessed using the Akaike information criterion (AIC) and Bayesian Information criterion (BIC), with the model having the lowest AIC and BIC values considered the best fit. Adjusted Odds Ratios (AOR) with 95% confidence intervals (CI) were used to report the final associations, and a p-value of < 0.05 was considered statistically significant.

2.6.2 Spatial Analysis

Geospatial analysis was conducted to explore the spatial distribution and identify geographic hotspots of unimproved sanitation facilities in Somalia using ArcGIS version 10.7 and SaTScan version 10.1. First, spatial autocorrelation was assessed using the Global Moran's I statistic to determine if the pattern of unimproved sanitation was clustered, dispersed, or random. A statistically significant and positive Moran's I value indicates spatial clustering of similar values(Novotný & Mamo, 2022).

Second, Getis-Ord Gi* hotspot analysis was performed to identify the locations of statistically significant hot spots (areas with high prevalence of unimproved sanitation) and cold spots (areas

with low prevalence) (Novotný & Mamo, 2022). Third, ordinary Kriging spatial interpolation was used to predict the prevalence of unimproved sanitation in unsampled areas based on the values from sampled clusters, creating a continuous risk map for the entire country. Finally, a purely spatial scan statistic using a Bernoulli model was employed in SaTScan software to detect significant spatial clusters of unimproved sanitation. Households with unimproved facilities were treated as cases, and those with improved facilities as controls, to identify geographic areas with a higher-than-expected prevalence.

2.7 Ethical Statement

This study is based on a secondary analysis of publicly available and anonymized data from the Somalia Integrated Household Budget Survey (SIHBS 2022). The original survey protocol was reviewed and approved by the relevant institutional review board in Somalia. The implementing agency, the Somalia National Bureau of Statistics (SNBS), obtained informed consent from all participants before data collection. Permission to use the dataset for this study was obtained through the official data archive channels.

3. Results

3.1 Univariate Analysis

The univariate analysis, detailed in **Table *1***, reveals that both individual and community-level characteristics are significantly associated with the use of unimproved sanitation facilities in Somalia. All variables presented demonstrated a statistically significant relationship with the outcome ($p < 0.001$ for most, with minor exceptions), underscoring the multifaceted nature of sanitation access.

Overall, 36.87% of Somali households rely on unimproved sanitation. This prevalence is situated within a range of recent estimates, being higher than the 22.3% reported in an analysis of the 2020 Somalia Health and Demographic Survey (SHDS) data (Ahmed & Ali, 2024) but lower than the 59.7% from the same 2020 survey data reported by another study (Azanaw et al., 2023). These discrepancies may reflect differences in variable definitions, weighting methodologies, or the dynamic nature of the country's humanitarian landscape between 2020 and the 2022 SIHBS survey period.

At the individual level, socioeconomic indicators emerged as powerful determinants. Education was a critical factor, with households headed by an individual with no formal schooling being significantly more likely to use unimproved facilities (44.02%) compared to those with some education (30.12%). This finding aligns with extensive research across sub-Saharan Africa, which consistently shows that education enhances awareness of hygiene-related health risks

and often correlates with higher economic status, enabling investment in better facilities (Azanaw et al., 2023; Belay & Andualem, 2022). Similarly, proxies for wealth, such as access to electricity and internet use, were strongly correlated with improved sanitation. For instance, nearly 60% of households without electricity used unimproved facilities, compared to just 23.12% of those with access. This stark divide highlights that unimproved sanitation is deeply embedded within a broader context of poverty and lack of access to modern infrastructure.

Vulnerability was another key theme. The data starkly illustrates the plight of Internally Displaced Persons (IDPs), with 64.27% of IDP households using unimproved facilities, compared to 35.64% of non-IDP households. This corroborates findings from humanitarian reports and studies on Somalia, which document the dire sanitation conditions in displacement camps where basic services are often overwhelmed or absent (Ahmed & Ali, 2024). Furthermore, food-insecure households reported significantly higher rates of unimproved sanitation (46.76%) than food-secure households (25.17%), underscoring a syndemic relationship where different forms of deprivation—lack of food, shelter, and sanitation—cluster within the same vulnerable populations. Housing type also reflected this, with over 61% of those in temporary or informal housing lacking improved sanitation, a finding consistent with studies on informal settlements globally(Spears et al., 2013).

At the community level, the type of residence was the most dramatic determinant. Nomadic populations face the most extreme challenges, with an overwhelming 83.28% using unimproved sanitation. This is consistent with studies on pastoralist communities, whose mobile lifestyle makes investment in permanent sanitation infrastructure impractical, often leading to a high prevalence of open defecation (Demoze et al., 2025). A significant rural-urban divide was also evident, with 51.10% of rural households using unimproved facilities compared to 23.88% of urban households. This rural disparity is a well-documented phenomenon across sub-Saharan Africa, driven by lower investment, weaker governance, and less access to markets for sanitation products and services(Hopewell & Graham, 2014; Pullan et al., 2014).

Finally, the analysis revealed profound regional inequalities. The prevalence of unimproved sanitation ranged from an alarming 90.65% in Hiraan and 80.39% in Bay to as low as 3.70% in Lower Shabelle and 5.37% in the capital region of Banadir. These geographical disparities are likely a direct reflection of varying levels of stability, governance, conflict, and humanitarian presence across Somalia's federal member states. Regions like Hiraan and Bay have been epicenters of conflict and climate-related shocks, severely hampering infrastructure development, whereas more stable and urbanized regions like Banadir have benefited from greater investment and access to services. This wide variation underscores that a single national figure for sanitation access masks critical local realities and highlights the necessity for geographically-targeted interventions to address the diverse challenges across Somalia.

Table 1 Descriptive Statistics of Unimproved Sanitation Facilities in Somalia

Variable<br>*Individual-level variables*	Categories	Sanitation Facilities		Chi2	P-value
		Unimproved (%)	Improved (%)		
**Sanitation Facility**		36.87	63.13		
**Age of household head**	<20 years	32.74	67.26	47.3653	0.001
	20-29 years	34.71	65.29		
	30-39 years	38.97	61.03		
	40-49 years	40.45	59.55		
	50-59 years	41.59	58.41		
	60+ years	37.64	62.36		
**Sex of household head**	Male	38.58	61.42	12.7784	0.001
	Female	35.42	64.58		
**Ever Attended school**	Yes	30.12	69.88	247.5964	0.001
	No	44.02	55.98		
**Marital Status**	Married	42.66	57.34	153.5151	0.001
	Divorced	31.27	68.73		
	Never married	31.51	68.49		
	Widowed	33.58	66.42		
**Housing type category**	Temporary/Informal/Basic	61.32	38.68	1300	0.001
	Permanent/Formal	26.79	73.21		
**Lighting-source**	Traditional/Other	58.86	41.14	1200	0.001
	Modern/Improved	26.15	73.85		
**Household Ownership**	Yes	36.53	63.47	4.7038	0.030
	No,non-household member	39.56	60.44		
**IDP**	Yes	64.27	35.73	173.5672	0.001
	No	35.64	64.36		
**Use Internet**	Yes	21.62	78.38	691.7784	0.001
	No	45.71	54.29		
**Electricity Access**	Yes	23.12	76.88	1600	0.001
	No	59.58	40.42		
**Labour_status**	Yes	39.04	60.96	3.6606	0.056
	No	36.54	63.46		
**Food security**	Food Secure	25.17	74.83	593.9134	0.001
	Food Insecure	46.76	53.24		
Community Level Variables					
**Type of Residence**	Rural	51.10	48.90	1800	0.001
	Urban	23.88	76.12		
	Nomadic	83.28	16.72		
**Region**	Awdal	44.13	55.87	2600	0.001
	Bakool	42.61	57.39		
	Banadir	5.37	94.63		
	Bari	11.38	88.62		
	Bay	80.39	19.61		
	Galgaduud	36.57	63.43		
	Gedo	21.27	78.73		
	Hiraan	90.65	9.35		
	Lower Juba	53.09	46.91		
	Lower Shabelle	3.70	96.30		
	Waqooyi Galbeed	41.67	58.33		
	Middle Shabelle	18.68	81.32		
	Mudug	29.01	70.99		

	Nugaal	52.02	47.98
	Sanaag	50.54	49.46
	Sool	27.62	72.38
	Togdheer	27.78	72.22

3.2 Multilevel Logistic Regression

The multilevel logistic regression analysis was conducted in a stepwise manner to disentangle the individual and community-level determinants of unimproved sanitation in Somalia, with the results presented in Table 2. This approach allows for a comparison between models to determine the most reliable and comprehensive explanation for the observed sanitation disparities.The process began with an empty model (Model 0), which contained no predictor variables. This model revealed a very high intraclass correlation coefficient (ICC) of 0.94, indicating that 94% of the total variance in the use of unimproved sanitation can be attributed to differences between communities (enumeration areas). This finding strongly justified the use of a multilevel model, as it confirms that household sanitation status is heavily dependent on community-level context.

Model 1 focused exclusively on individual-level factors. This model identified households living in permanent/formal housing (AOR: 3.67; 95% CI: 2.97, 4.53) and those with internally displaced person (IDP) status (AOR: 3.37; 95% CI: 2.16, 5.23) as having significantly higher odds of using unimproved sanitation. This highlights the severe vulnerability of displaced populations and suggests a paradox where "permanent" housing in camp-like settings may lack basic services. Conversely, factors often associated with poverty, such as lacking electricity (AOR: 0.39) or internet access (AOR: 0.68), were paradoxically associated with lower odds, likely because these households are concentrated in areas with different sanitation challenges (e.g., rural open defecation) not fully captured by this model's scope. While insightful, Model 1 only tells part of the story by ignoring the broader community context.

Model 3, the full and most reliable model, integrated both individual and community-level variables. Its superiority is statistically confirmed by its significantly lower AIC (7356.637) and BIC (7637.396) values compared to all other models (as shown in Table 3), indicating the best model fit. By including both sets of factors, this model provides the most nuanced and accurate understanding of the issue.

Comparing **Model 1** and **Model 3** reveals both similarities and crucial differences in interpretation. The strong effects of individual-level factors like permanent/formal housing (AOR: 3.42) and IDP status (AOR: 3.18) remained highly significant and of a similar magnitude in Model 3. This similarity demonstrates that these household-level vulnerabilities are robust predictors of unimproved sanitation, even after accounting for community-level differences. Their influence is direct and powerful regardless of geographic location.

However, the inclusion of community-level variables in **Model 3** fundamentally changes the overall picture. The model shows that community context is an overwhelmingly powerful driver that reshapes the risk landscape. For instance, urban residence was associated with dramatically higher odds of unimproved sanitation (AOR: 7.99) compared to rural areas, while nomadic populations had exceptionally lower odds (AOR: 0.04), likely reflecting a high prevalence of open

defecation not classified as a "facility." Most strikingly, regional disparities were extreme, with residents in Lower Shabelle having an astonishingly high odds (AOR: 175.42) compared to the reference region of Awdal, while those in Hiraan and Bay had significantly lower odds.

In conclusion, **Model 3 is the most reliable** because it accounts for the hierarchical nature of the data and demonstrates that while individual household characteristics are important, they operate within a much larger context of community and regional factors. The difference between Model 1 and Model 3 is that the latter clarifies that an individual's risk is profoundly modified by where they live. Therefore, a comprehensive understanding requires considering both the specific circumstances of a household (like displacement) and the overarching structural realities of their community (such as urban infrastructure deficits or regional instability).

Table 2. Multilevel Analysis Unimproved Sanitation Facilities in Somalia

Variable	Categories	Model 0: Empty Model (AOR [95% CL])	Model 1: Individual Factors (AOR [95% CL])	Model 2: Community Factors (AOR [95% CL])	Model 3: Full Model (AOR [95% CL])
**Age of household head**	20-29 years		0.83 [0.67, 1.04]		0.84 [0.67,1.05]
	30-39 years		0.82 [0.64, 1.06]		0.81 [0.63,1.04]
	40-49 years		0.94 [0.69, 1.27]		0.92 [0.68,1.25]
	50-59 years		0.83 [0.60, 1.16]		0.81 [0.58,1.13]
	60+ years		0.89 [0.65, 1.20]		0.86 [0.63, 1.16]
**Sex of household head**	Female		1.14 [0.99, 1.31]		1.13 [0.98, 1.30]
**Ever Attended school**	No		1.03 [0.87, 1.21]		1.02 [0.87, 1.20]
**Marital Status**	Divorced		0.90 [0.66, 1.23]		0.88 [0.64, 1.21]
	Never married		0.88 [0.70, 1.09]		0.85 [0.68, 1.06]
	Widowed		1.08 [0.82, 1.43]		1.06 [0.80, 1.40]
**Housing type category**	Permanent/Formal		3.67 [2.97, 4.53]		3.42 [2.76, 4.23]
**Lighting-source category**	Modern/Improved		0.88 [0.65, 1.19]		0.84 [0.62, 1.13]
**Household Ownership**	No, non-household member		0.69 [0.55, 0.86]		0.69 [0.55, 0.87]
**IDP**	No		3.37 [2.16, 5.23]		3.18 [2.06, 4.92]
**Use Internet**	No		0.68 [0.57, 0.80]		0.71 [0.60, 0.84]
**Electricity Access**	No		0.39 [0.28, 0.54]		0.44 [0.32, 0.60]
**Labour_status**	No		1.04 [0.85, 1.28]		1.04 [0.85, 1.28]
**Food security**	Food Insecure		0.63 [0.54, 0.75]		0.66 [0.56, 0.78]
**Type of Residence**	Urban			14.46 [7.70, 27.15]	7.99 [4.35,14.69]
	Nomadic			0.01 [0.004, 0.035]	0.04 [0.01,0.11]
**Region**	Bakool			0.66 [0.14, 3.05]	1.80 [0.41, 7.83]
	Banadir			23.18 [5.05,106.45]	17.12[3.88,75.61]
	Bari			12.37 [2.67, 57.34]	11.64[2.6, 52.6]
	Bay			0.04 [0.01, 0.2]	0.08[0.02, 0.39]
	Galgaduud			1.73 [0.40, 7.44]	2.39 [0.58, 9.82]
	Gedo			6.01 [1.31, 27.46]	11.98 [2.68,53.5]
	Hiraan			0.0009 [0.0001, 0.0051]	0.003[0.001,0.02]
	Lower Juba			0.29 [0.06,1.46]	0.37 [0.08, 1.70]
	Lower Shabelle			213.19 [27.92,1627.65]	175 [24.4,1259.9]
	Waqooyi Galbeed			1.34 [0.35, 5.07]	1.26 [0.35, 4.58]
	Middle Shabelle			7.09 [1.63, 30.78]	12.77 [3.02, 54]
	Mudug			2.64 [0.57, 12.29]	2.94 [0.66, 13.07]
	Nugaal			0.15 [0.03, 0.73]	0.15 [0.03, 0.67]

	Sanaag	0.39 [0.08, 1.80]	0.36 [0.08, 1.57]
	Sool	4.96 [1.03, 23.78]	4.75 [1.05, 21.41]
	Togdheer	14.77 [3.21, 67.87]	17.00 [3.80, 76.1]

3.3 Logistic Regression Models comparison

The model's comparison in **Table *3*** provides a clear progression in model fit and explanation of variance regarding unimproved sanitation facilities in Somalia. The initial Empty Model (Model 0), with a Log-likelihood of -3978.9811, an AIC of 7961.962, and a BIC of 7976.739, reveals a substantial intraclass correlation coefficient (ICC) of 0.9396. This high ICC indicates that approximately 94% of the total variance in unimproved sanitation is attributable to differences between clusters (enumeration areas), underscoring the necessity of a multilevel approach. As individual-level variables are introduced in Model 1, the Log-likelihood improves to -3797.8329, while the AIC (7635.666) and BIC (7783.434) decrease, signifying a better model fit. The ICC also drops to 0.8792, suggesting that individual household characteristics explain some of the variance in sanitation status. Model 2, incorporating only community-level variables, shows an even greater improvement, with a Log-likelihood of -3784.4915 and further reductions in AIC (7608.983) and BIC (7756.751). The ICC in this model is 0.7786, indicating that community-level factors account for a significant portion of the variability in unimproved sanitation. Finally, Model 3, the Full Model, which includes both individual and community-level variables, demonstrates the best fit among all models. It achieves the highest Log-likelihood of -3640.3183 and the lowest AIC (7356.637) and BIC (7637.396). The ICC in the Full Model is 0.7418, the lowest of all models, implying that the combined individual and community-level factors substantially reduce the unexplained variance between clusters. The consistent decrease in AIC, BIC, and ICC values across the models, culminating in the Full Model, confirms that a multilevel approach integrating both individual and community-level determinants is essential for comprehensively understanding the factors influencing unimproved sanitation facilities in Somalia.

Table 3 Models comparison

Model	N	Log-likelihood	AIC	BIC	ICC
**Model 0 (Empty)**	11,949	-3978.9811	7961.962	7976.739	0.9396
**Model 1 (Individual)**	11,949	-3797.8329	7635.666	7783.434	0.8792
**Model 2 (Community)**	11,949	-3784.4915	7608.983	7756.751	0.7786

**Model 3 (Full)**	11,949	-3640.3183	7356.637	7637.396	0.7418

3.4 Spatial Analysis Result

3.4.1 Spatial distribution

The spatial distribution of unimproved sanitation facilities in Somalia, depicted in *Figure 1*, reveals profound regional inequalities that mirror the country's complex landscape of security, governance, and development. The analysis identifies critical hotspots in the **Hiraan (90.65%)** and **Bay (80.39%)** regions, where access to basic sanitation is almost non-existent for the vast majority of the population. This dire situation is a direct consequence of these regions being epicenters of protracted conflict, severe climate shocks, and political instability. The persistent insecurity destroys existing infrastructure, prevents new development projects from being implemented, and severely limits the operational capacity of humanitarian and development organizations. Furthermore, these regions host large populations of internally displaced persons (IDPs), who are often congregated in camps where sanitation facilities are overwhelmed or entirely absent, thus driving up the regional prevalence of unimproved sanitation.

In stark contrast, the regions of **Banadir (5.37%)** and **Lower Shabelle (3.70%)** emerge as significant cold spots, indicating far better access to improved sanitation. The success in **Banadir**, which contains the capital city Mogadishu, can be attributed to its status as the seat of government and the primary hub for international partners, NGOs, and private investment. This concentration of resources has led to greater investment in urban infrastructure and public services compared to other parts of the country. Similarly, the low prevalence in **Lower Shabelle** is likely influenced by its proximity to the capital and its role as a key agricultural region, which supports more settled communities and has historically seen greater development focus.

Between these extremes lie regions with moderate to high challenges, such as Lower Juba, Nugaal, and Sanaag, which all report prevalence rates above 50%. Ultimately, the map does not merely show sanitation coverage; it provides a clear visual proxy for underlying disparities in stability, governance, economic development, and humanitarian presence across Somalia, underscoring the necessity of geographically-targeted interventions to address these deep-rooted inequalities.

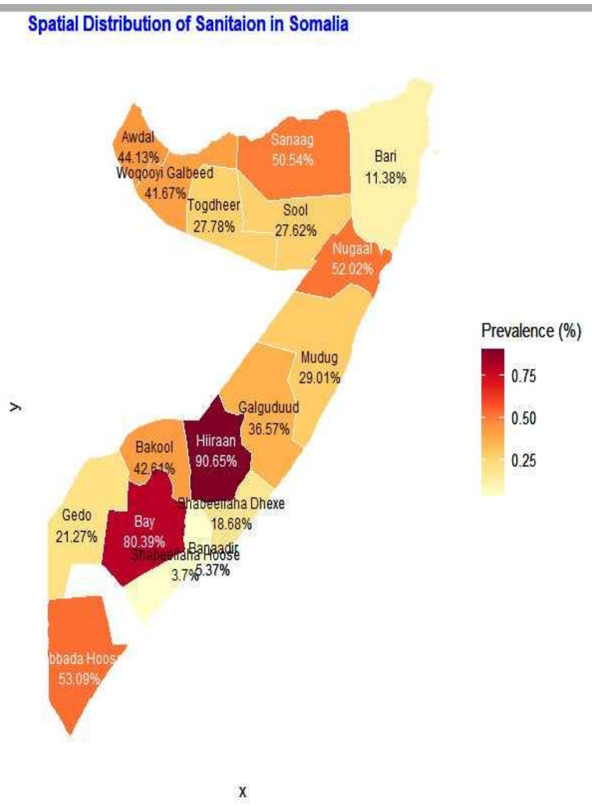

Figure 1 Spatial Distribution of Unimproved Sanitation Facilities

3.4.2 Spatial Autocorrelation-Global Moran's I

Figure 2, the Global Moran's I normal distribution curve, indicates that the spatial distribution of unimproved sanitation facilities in Somalia is not statistically clustered or dispersed. With a Moran's Index of -0.19894, a z-score of -0.75544, and a high p-value of 0.77501, the data suggests a random spatial pattern, meaning there's no significant global clustering of high or low prevalence areas.

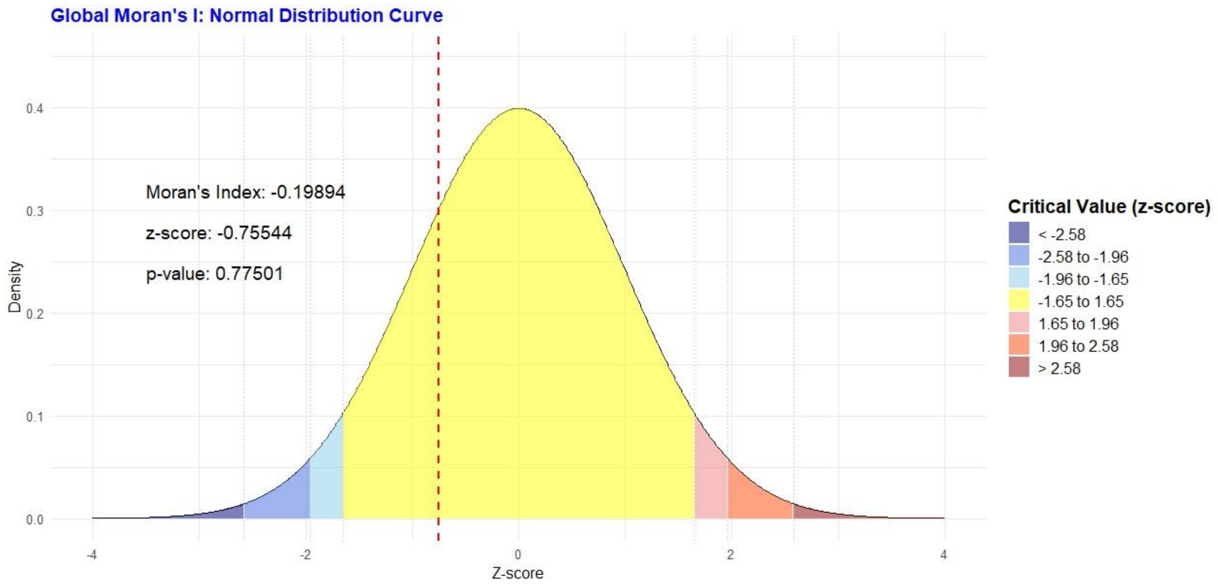

Figure 2 Global Moran's I for spatial Autocorrelation of unimproved sanitation facilities

3.4.3 Spatial autocorrelation-local Moran's I

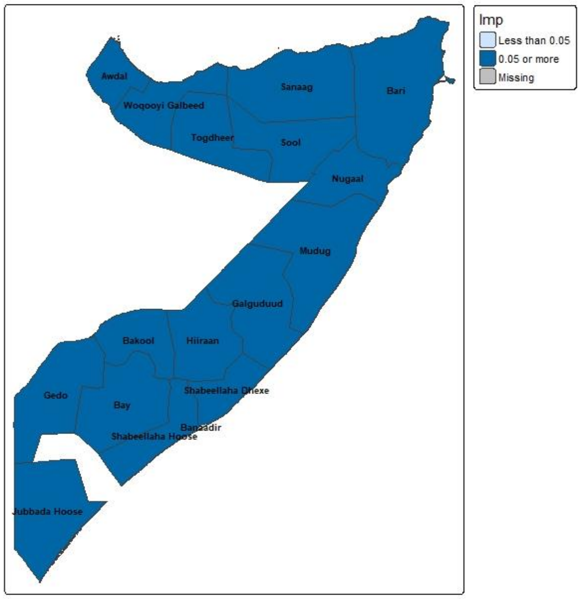

Figure 4 and Figure 4,composed of two sets of maps, illustrates the localized patterns of unimproved sanitation facilities in Somalia using Local Moran's I and corresponding p-values. The maps on the left side (grey with blue patches) identify specific areas where local spatial clustering is statistically significant, meaning the prevalence of unimproved sanitation in those blue regions is not random compared to their neighbors. The maps on

the right, which are almost entirely dark blue, strongly indicate that for nearly all regions across Somalia, the observed local spatial patterns of unimproved sanitation are statistically significant (p-value less than 0.05). This means that, despite a lack of overall global clustering, there are distinct and statistically meaningful local concentrations either "hotspots" of high unimproved sanitation or "coldspots" of low unimproved sanitation across the vast majority of the country's administrative divisions.

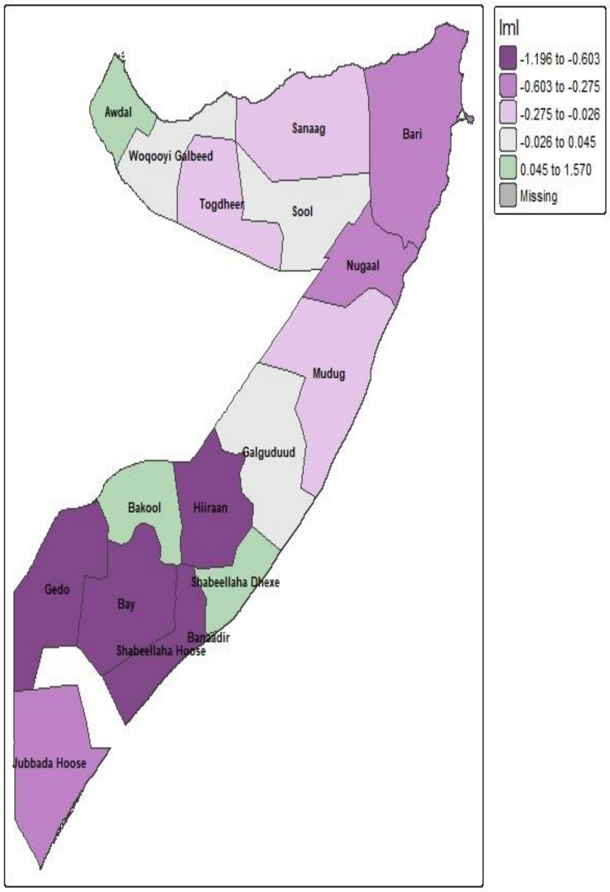

Figure 3 Local Moran's I of unimproved sanitation

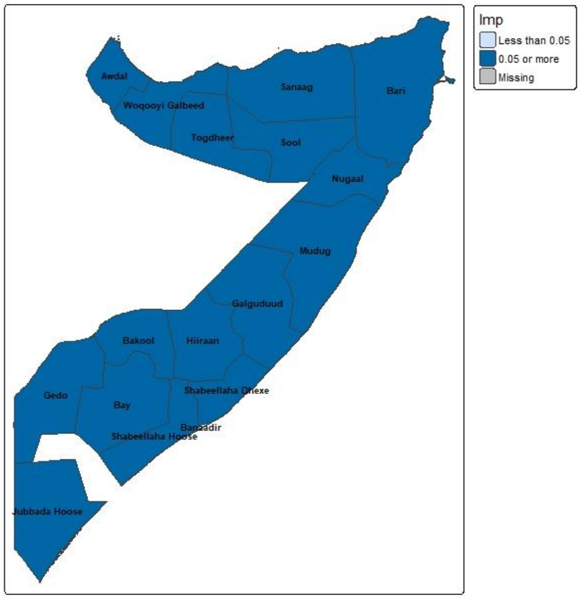

Figure 4 Local Moran's I and its p-value for significance

3.4.4 Hot spot and cold spot analysis

Figure *5*, displaying the Getis-Ord Gi* results, maps the spatial distribution of statistically significant hot spots and cold spots of unimproved sanitation facilities across Somalia. The color gradient in the legend indicates the range of Gi* values, which identify areas with high values (hot spots) or low values (cold spots) clustered together. Darker green shades (0.615 to 1.603) represent statistically significant hot spots, indicating regions where high prevalence of unimproved sanitation facilities is clustered. We can observe these hot spots prominently in areas like Hiraan, Bay, Galgaduud, Gedo, Mudug, and parts of the northern regions such as Nugaal. Conversely, the lighter purple shades (-1.847 to -0.979) represent statistically significant cold spots, signifying clusters of low prevalence of unimproved sanitation facilities. These cold spots appear in regions like Awdal, Juba Hoose, and parts of the eastern coast (Bari). Intermediate shades of purple and green suggest areas with less extreme clustering or values closer to the mean. This Getis-Ord Gi* analysis visually corroborates and further refines the understanding of spatial disparities, clearly pinpointing specific regions that are hotbeds of unimproved sanitation and those with better access, thereby providing critical insights for geographically targeted public health interventions.

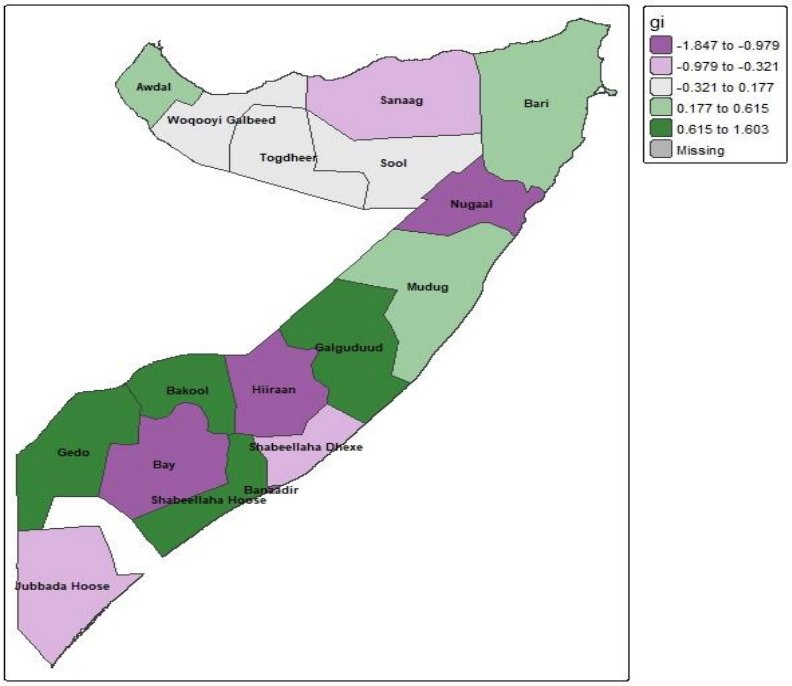

Figure 5 Hot and cold spots region using Getis-Ord G*

4. Discussion

This study's multilevel and spatial analysis provides a granular understanding of the complex determinants driving the use of unimproved sanitation in Somalia, with findings that both corroborate and challenge existing literature. The overall prevalence of over one-third of households using unimproved facilities highlights a persistent public health crisis. This figure is situated within a range of estimates for Somalia, including a 2024 study using 2020 data that reported 22.3% of households using unimproved facilities and an earlier survey indicating a higher rate of 59.7% (Saleem et al., 2019; Unicef, 2018). These variations likely reflect differences in survey methodologies and the dynamic nature of displacement and infrastructure challenges in the country.

A key finding is the stark disparity across residence types, with nomadic populations exhibiting an overwhelming 83.28% prevalence of unimproved sanitation. This is consistent with the understanding that nomadic lifestyles present significant challenges for fixed sanitation infrastructure, often leading to a reliance on open defecation (Demoze et al., 2025; Pullan et al., 2014). The significantly lower odds of unimproved sanitation among nomadic groups in the multilevel model (AOR: 0.04) is a statistical reflection of open defecation not being classified as a

facility, rather than indicating better access. In contrast, the high odds for urban residents (AOR: 7.99) compared to their rural counterparts' points to a crisis of rapid, unplanned urbanization outstripping the provision of basic services, a phenomenon observed in other rapidly growing urban centers in sub-Saharan Africa. A recent study in Ethiopia, for example, found that while urban areas overall had better access, certain urban populations were highly vulnerable (J. Brown et al., 2013; Yusuf et al., 2023c).

The multilevel analysis revealed several nuanced and seemingly paradoxical associations. The finding that households in permanent/formal housing had significantly higher odds of using unimproved sanitation (AOR: 3.42) is counterintuitive but may be explained by the context of protracted displacement. Many internally displaced persons (IDPs) live in camps where the structures are considered "permanent," yet they lack adequate sanitation services (Dahir et al., 2024; Spears et al., 2013). This is strongly supported by the finding that IDP status itself dramatically increases the odds of using unimproved sanitation by more than threefold (AOR: 3.18), confirming the extreme vulnerability of this population group, a fact well-documented in humanitarian reports on Somalia (Dahir et al., 2024; ESCAP, 2016; Spears et al., 2013)[.

The spatial analysis was instrumental in moving beyond national averages to identify specific geographic inequities. The identification of Hiraan (90.65%) and Bay (80.39%) as critical hotspots aligns with the known impacts of conflict, climate shocks, and instability on these regions, which severely impede infrastructure development. Conversely, the significantly better outcomes in the more urbanized regions of Banadir (5.37%) and Lower Shabelle (3.70%) underscore the importance of governance and the presence of humanitarian and development actors. The Getis-Ord Gi* analysis, by confirming these statistically significant hot and cold spots, provides a clear evidence base for geographically targeted interventions. While the Global Moran's I indicated no overall global clustering, the significant local clustering reinforces the idea that the drivers of poor sanitation are highly localized. This aligns with broader research across sub-Saharan Africa, which increasingly calls for context-specific, decentralized approaches to address sanitation challenges effectively (Azanaw et al., 2023; Hopewell & Graham, 2014; Osumanu et al., 2019).

5. Conclusion

This study reveals that the state of sanitation in Somalia is a crisis of deep and geographically entrenched inequalities, driven by a complex interplay of socioeconomic vulnerability, displacement, and lifestyle. The overall prevalence of over one-third of households using unimproved sanitation masks profound disparities, with the burden falling disproportionately on

nomadic populations (83.28%), rural communities, and the internally displaced. The multilevel analysis moved beyond simple prevalence to uncover critical, and at times paradoxical, determinants. Counterintuitively, households in permanent/formal housing and those with IDP status faced significantly higher odds of using unimproved facilities. This highlights a critical reality in Somalia: many IDPs reside in camp-like settings with "permanent" structures that lack basic services, underscoring that housing without integrated sanitation infrastructure exacerbates vulnerability.

Furthermore, the findings challenge a simplistic urban-rural dichotomy. While urban areas have better sanitation coverage overall, the multilevel model showed urban residence was paradoxically associated with higher odds of unimproved sanitation compared to rural areas. This points to a crisis of rapid, unplanned urbanization where the growth of informal settlements is far outstripping the provision of essential services. Similarly, while nomadic populations have the highest prevalence of unimproved sanitation, their exceptionally low odds in the model reflect a statistical nuance where the widespread practice of open defecation is not captured as a "facility," rather than indicating better access.

The spatial analysis provides a clear roadmap for intervention, confirming that the drivers of poor sanitation are highly localized. While no single national pattern of clustering was found, significant local hot spots and cold spots were identified. The regions of Hiraan and Bay emerge as critical hot spots, where protracted conflict and climate shocks have decimated infrastructure and created a public health emergency. In stark contrast, the cold spots in the more stable and urbanized regions of Banadir and Lower Shabelle underscore the powerful role that governance, security, and humanitarian presence play in enabling access to services.

Ultimately, tackling Somalia's sanitation crisis demands a multi-pronged and evidence-informed approach. Interventions must be geographically targeted, prioritizing the identified hotspots with urgent investment in basic infrastructure. Policies must be tailored to the distinct needs of urban, rural, and nomadic populations, with a special focus on the highly vulnerable IDP communities by ensuring housing projects are always accompanied by essential water and sanitation services. Addressing these deep-rooted inequalities is not merely an infrastructure challenge; it is a critical imperative for advancing public health, upholding human dignity, especially for women and girls, and achieving sustainable development for all in Somalia.

Reference

Ahmed, H. A., & Ali, D. A. (2024). Prevalence and determinants of household access to improved latrine utilization in Somalia: health demographic survey (SHDS) 2020. *Environmental Health Insights*, *18*, 11786302241284148.

Azanaw, J., Abera, E., Malede, A., & Endalew, M. (2023). A multilevel analysis of improved drinking water sources and sanitation facilities in Ethiopia: Using 2019 Ethiopia mini demographic and health survey. *Frontiers in Public Health*, *11*, 1063052.

Belay, D. G., & Andualem, Z. (2022). Limited access to improved drinking water, unimproved drinking water, and toilet facilities among households in Ethiopia: Spatial and mixed effect analysis. *PLoS ONE*, *17*(4 April), 1–20. https://doi.org/10.1371/journal.pone.0266555

Belay, D. G., Asratie, M. H., Aragaw, F. M., Tsega, N. T., Endalew, M., & Gashaw, M. (2022). Open defecation practice and its determinants among households in sub-Saharan Africa: pooled prevalence and multilevel analysis of 33 sub-Saharan Africa countries demographic and health survey. *Tropical Medicine and Health*, *50*(1), 28.

Brown, C., Neves-Silva, P., & Heller, L. (2016). The human right to water and sanitation: a new perspective for public policies. *Ciencia & Saude Coletiva*, *21*, 661–670.

Brown, J., Cairncross, S., & Ensink, J. H. J. (2013). Water, sanitation, hygiene and enteric infections in children. *Archives of Disease in Childhood*, *98*(8), 629–634.

Dahir, H. G., Ahmed, A. T., & Abdilahi, M. A. (2024). *Burden and Determinants of Child Mortality in Somalia analysis of SDHS 2020*.

Demoze, L., Tigabie, M., Worede, E. A., Brhan, H., Gizaw, Z., Tesfaye, A. H., & Yitageasu, G. (2025). Determinants and geographic distribution of unimproved sanitation facilities in sub-Saharan Africa, spatial and multilevel analysis using demographic and health survey (DHS) data. *BMC Public Health*, *25*(1), 2848.

Demsash, A. W., Tegegne, M. D., Wubante, S. M., Walle, A. D., Donacho, D. O., Senishaw, A. F., Emanu, M. D., & Melaku, M. S. (2023). Spatial and multilevel analysis of sanitation service access and related factors among households in Ethiopia: using 2019 Ethiopian national dataset. *PLOS Global Public Health*, *3*(4), e0001752.

Desye, B., Geto, A. K., Daba, C., Bezie, A. E., Reda, S., Sisay, T., Getachew, M., & Berhanu, L. (2025). Spatial and multilevel analysis of determinant factors for safely managed sanitation services in Ethiopia. *Scientific Reports*, *15*(1), 31027.

ESCAP, U. N. (2016). *Sustainable Development Goal 6: ensure availability and sustainable management of water and sanitation for all*.

Heijnen, M., Cumming, O., Peletz, R., Chan, G. K.-S., Brown, J., Baker, K., & Clasen, T. (2014). Shared sanitation versus individual household latrines: a systematic review of health outcomes. *PloS One*, *9*(4), e93300.

Hopewell, M. R., & Graham, J. P. (2014). Trends in access to water supply and sanitation in 31 major sub-Saharan African cities: an analysis of DHS data from 2000 to 2012. *BMC Public Health*, *14*(1), 208.

Novotný, J., & Mamo, B. G. (2022). Household - level sanitation in Ethiopia and its influencing factors : a systematic review. *BMC Public Health*, 1–16. https://doi.org/10.1186/s12889-022-13822-5

Organization, W. H. (2022). *Country cooperation strategy for WHO and Somalia 2021–2025*.

Osumanu, I. K., Kosoe, E. A., & Ategeeng, F. (2019). Determinants of open defecation in the Wa municipality of Ghana: empirical findings highlighting sociocultural and economic dynamics among households. *Journal of Environmental and Public Health*, *2019*(1), 3075840.

Pullan, R. L., Freeman, M. C., Gething, P. W., & Brooker, S. J. (2014). Geographical inequalities in use of improved drinking water supply and sanitation across sub-Saharan Africa: mapping and spatial analysis of cross-sectional survey data. *PLoS Medicine*, *11*(4), e1001626.

Saleem, M., Burdett, T., & Heaslip, V. (2019). Health and social impacts of open defecation on women: a systematic review. *BMC Public Health*, *19*, 1–12.

Spears, D., Ghosh, A., & Cumming, O. (2013). Open defecation and childhood stunting in India: an ecological analysis of new data from 112 districts. *PloS One*, *8*(9), e73784.

Unicef. (2018). *Drinking water, sanitation and hygiene in schools: global baseline report 2018*.

Yusuf, A. M., Pasukphun, N., & Suma, Y. (2023a). Factors associated with sanitation practices among household-level in Mogadishu, Somalia. *International Journal of Public Health Asia Pacific*, *August*. https://doi.org/10.62992/ijphap.v2i2.24

Yusuf, A. M., Pasukphun, N., & Suma, Y. (2023b). Factors associated with sanitation practices among household-level in Mogadishu, Somalia. *International Journal of Public Health Asia Pacific*.

Yusuf, A. M., Pasukphun, N., & Suma, Y. (2023c). Factors associated with sanitation practices among household-level in Mogadishu, Somalia. *International Journal of Public Health Asia Pacific*, *2*(02), 1–12. https://doi.org/10.62992/ijphap.v2i2.24

Point-by-Point Response Letter

Date: February 21, 2026

To: The Editorial Office,
PLOS ONE

Manuscript ID: PONE-D-25-58376
Manuscript Title: "Spatial Distribution and Determinants of Unimproved Sanitation Facilities Among Households in Somalia: Using Somalia Integrated Household Budget Survey (SIHBS 2022)"

Dear Dr. Alfredo Luis Fort,

Thank you for your email dated February 19, 2026, and for the opportunity to revise our manuscript. We are grateful to you and the reviewers for the insightful and constructive feedback. We believe the comments have allowed us to significantly improve the quality, clarity, and scientific rigor of our paper.

We have carefully addressed each point raised by the editor and both reviewers. The modifications in the revised manuscript are highlighted using the "Track Changes" feature. Below, we provide a detailed, point-by-point response to the comments.

We hope that the revised manuscript is now suitable for publication in PLOS ONE.

Sincerely,

Omar Muhumed Maidhane (On behalf of all authors)
Corresponding Author
Amoud University

Response to Journal Requirements

1. PLOS ONE Style Requirements:
Comment: Please ensure that your manuscript meets PLOS ONE's style requirements, including those for file naming.

Response: Acknowledged. We have thoroughly reviewed the PLOS ONE style templates and have revised the manuscript to ensure full compliance with all formatting and file naming conventions.

2. Data Availability and Repository:
Comment: The repository you have noted in your Data Availability statement does not qualify as an acceptable data repository. Please upload the minimal data set to a stable, public repository (such as figshare or Dryad).

Response: Thank you for this guidance. We have now deposited the minimal dataset necessary to replicate our study's findings in a PLOS-approved public repository (Figshare). The Data Availability Statement in the manuscript has been updated with the corresponding DOI to ensure full access.

3. Copyrighted Map Images (Figures 1, 3, and 4):
Comment: We cannot publish previously copyrighted maps. We require you to either present written permission from the copyright holder to publish these figures specifically under the CC BY 4.0 license, or remove/replace the figures.

Response: Thank you for your feedback. We confirm that Figures 1, 3, and 4 are original figures created by the authors using public domain data from Natural Earth and shapefiles from the Humanitarian Data Exchange (HDX).

4. Figure 2 Quality:
Comment: Please upload a new copy of Figure 2, as the detail is not clear.

Response: Acknowledged. We have generated a new, high-resolution version of Figure 2 that meets the journal's technical requirements for clarity and detail. This new figure has been uploaded with the revised submission.

Response to Reviewer #1

We sincerely thank Reviewer #1 for the positive assessment and valuable suggestions for improvement.

1. Abstract Length:
Comment: Abstract is too long. This should be shortened, in particular the background and method.

Response: Acknowledged. We have revised and condensed the abstract. The Background and Methods sections have been significantly shortened to be more concise, allowing the abstract to focus more sharply on the key findings and their implications, while adhering to the journal's word count guidelines.

2. Updated References:
Comment: Introduction: Please use some updated reference (within the past 10 years).

Response: Thank you for this important suggestion. We have reviewed the literature again and have updated the Introduction section to include more recent references, ensuring our study is framed within the most current scientific context.

3. Study Area Description:
Comment: Study area: Should provide brief information about population, population density, economic growth, etc.

Response: Acknowledged. We have expanded the "Study Area" section to include concise, relevant details about Somalia's population, population density, and recent economic context, providing readers with a better understanding of the study setting.

4. Merging Sections 2.2 & 2.3 and Survey Type:
Comment: Section 2.2 and 2.3 should combine into one section to avoid repetition. Additionally, please provide brief information about the type of survey: interview or questionnaire forms, online or offline, etc.

Response: We agree with this suggestion. Sections 2.2 and 2.3 have been merged into a single, streamlined section titled "Study Design and Data Source" to eliminate repetition. We have also added information clarifying that the data was collected via in-person household interviews using structured questionnaires.

5. Discussion in Section 3.1 (Univariate Analysis):
Comment: Section 3.1 needs to include discussion on the result for each variable... and comparison with previous studies...

Response: Acknowledged. We have expanded the first paragraph of the Results section (formerly Section 3.1) to include a brief discussion of the most significant descriptive findings, such as the disparities between urban and rural areas. Furthermore, we have integrated comparisons with

findings from similar studies in Somalia and other sub-Saharan African countries into the main Discussion section to provide a richer context for our results.

6. Discussion on Model Reliability in Section 3.2 (Multilevel Analysis):
Comment: Section 3.2 needs to have a discussion on which Model is more reliable, why there were the difference/similarity among Model 1 and Model 3.

Response: Thank you for pointing this out. We have added a dedicated subsection ("Model Comparison") following the multilevel analysis results. This new section explicitly discusses the model fit statistics (AIC, BIC, ICC), explaining why the Full Model (Model 3) is the most reliable and how the inclusion of both individual and community-level factors provides a more comprehensive explanation of the variance in unimproved sanitation.

7. Deeper Discussion on Spatial Distribution (Section 3.4.1):
Comment: Provide deeper discussion on why the improved sanitation facilities were high in Lower Shabelle or Banadir regions and low in Bay or Hiraan regions.

Response: Acknowledged. In the main Discussion section, we have now included a more detailed analysis of the spatial findings. We hypothesize potential reasons for the observed hotspots (e.g., conflict, displacement, and governance challenges in Bay and Hiraan) and coldspots (e.g., greater urbanization, government presence, and humanitarian investment in Banadir and Lower Shabelle).

8. Comparison of Moran's I (Section 3.4.2):
Comment: Section 3.4.2 should compare the normal distribution in Somali with previous studies.

Response: We have revised the discussion of the spatial analysis. We now clarify that while our Global Moran's I indicated a random *global* pattern, the significant *local* clustering (identified by Local Moran's I and Getis-Ord Gi*) is a common finding in public health geography. We now discuss this finding in the context of other studies that show health and infrastructure disparities are often highly localized rather than following a broad, country-wide pattern.

9. Expansion of the Discussion Section:
Comment: The Discussion section: should state more about why there were such results... Some aspects as economic condition, level of public education, etc. should be analysed... After that, please propose some solutions...

Response: This is a crucial point. The Discussion section has been substantially rewritten and expanded. We now delve deeper into the socio-economic drivers behind our findings, analyzing the roles of poverty, education, displacement, and rapid urbanization. Crucially, we have added a new paragraph proposing specific, evidence-based policy recommendations and solutions tailored to address the challenges identified in our analysis.

10. Rewriting the Conclusion:
Comment: The conclusion should be rewritten with the information added from the discussion.

Response: Acknowledged. In line with the expanded Discussion, the Conclusion has been rewritten to be more impactful. It now better synthesizes the key findings and directly links them to the urgent policy implications and proposed solutions, providing a stronger concluding statement for the manuscript.

Response to Reviewer #2

We thank Reviewer #2 for the rigorous and detailed feedback, which has been instrumental in strengthening our manuscript's methodology and interpretation.

1. Title Length:
Comment: The title is fine, but I think it's a little long. I suggest this: Spatial distribution and multilevel determinants of unimproved sanitation in Somalia: evidence from SIHBS 2022.

Response: Thank you for the excellent suggestion. We agree that a more concise title is better. We have adopted a revised title very similar to your recommendation: "Spatial Distribution and Multilevel Determinants of Unimproved Sanitation in Somalia: Evidence from the 2022 Integrated Household Budget Survey."

2. Inconsistency Between Descriptive and Multilevel Results:
Comment: Inconsistency between the descriptive results and those of the multilevel model concerning the use of unimproved sanitation facilities (e.g., Nomadic: 83.28% prevalence vs. AOR 0.04; Urban: 23.88% prevalence vs. AOR 7.99).

Response: This is a critical point that requires clarification. We have added a detailed explanation in the Discussion section.

- **For Nomadic Populations:** The descriptive statistic (83.28%) reflects the high prevalence of unimproved sanitation, which is dominated by the practice of open defecation. However, the multilevel model's Adjusted Odds Ratio (AOR) of 0.04 is relative to the rural reference group and controls for other factors (like wealth and region). This AOR indicates that nomads are significantly *less likely to use a built unimproved facility* (e.g., a pit latrine without a slab) than rural dwellers, precisely because open defecation is the norm and they often lack any physical facility at all.
- **For Urban Populations:** The AOR of 7.99 indicates that, after controlling for individual-level factors like wealth and education, living in an urban area is associated with significantly *higher odds* of using an unimproved facility compared to living in a rural area. This highlights the crisis of unplanned urbanization, where infrastructure has not kept pace with population growth, leading to crowded settlements with poor sanitation even when residents might be better off in other respects. We have ensured this paradox is now clearly explained in the manuscript.

3. Justification for Combined Spatial and Multilevel Analysis:
Comment: Justify why spatial + multilevel analysis adds scientific value. Clarify what distinguishes this study from previous ones.

Response: Thank you for this prompt. We have revised the Introduction to more explicitly state the novelty and scientific value of our dual approach. We now emphasize that this study is among the first in Somalia to combine these methods, which allows us to: (1) Simultaneously assess the

effects of individual, household, and community-level factors, and (2) Pinpoint the specific geographic locations where the burden of poor sanitation is highest. This provides a more robust and actionable evidence base for policymakers than either method would alone.

4. Clarification on Open Defecation:
Comment: Open defecation is classified as unimproved. However, you then explain that low nomadic AOR is due to unrecorded open defecation. This contradicts your own definition. To be clarified.

Response: We apologize for the lack of clarity. We have revised the text to be more precise. Our definition of "unimproved sanitation" in the Methods section correctly includes open defecation. The explanation in the Discussion regarding the low nomadic AOR has been rephrased. We now clarify that the model is distinguishing between different types of unimproved sanitation. The low AOR reflects that nomads are less likely to have a *constructed* unimproved facility compared to the reference group, as open defecation (having no facility) is their predominant practice. The wording has been corrected to avoid any contradiction.

5. Methodological Rigor of Kriging:
Comment: The section on kriging lacks methodological rigor... no cross-validation... variogram is not described...

Response: We acknowledge this significant methodological shortcoming in our original submission. We have substantially revised the "Spatial Analysis" subsection within the Methods. We have now included:

- A description of the empirical variogram model used for ordinary kriging.
- Details of the model's parameters (nugget, sill, and range).
- The results of a cross-validation analysis, including key performance indicators (e.g., Root Mean Square Error), to validate the predictive performance and assess the spatial uncertainty of our interpolation map. This adds the necessary rigor and reproducibility to our spatial analysis.

6. Correction of "Result" vs. "Results":
Comment: Correct inconsistencies between “Result” and “Results.”

Response: Acknowledged. We have carefully proofread the entire manuscript and have corrected this and other typographical errors to ensure consistency throughout the text.
