# Supplementary material for: Spatial distribution and determinants of unimproved sanitation facilities among households in Somalia: Using Somalia integrated household budget survey (SIHBS 2022)
Source: PLoS One. 2026 Apr 24;21(4):e0348029. doi: 10.1371/journal.pone.0348029 (PMC13108892; doi:10.1371/journal.pone.0348029)
Supplement: Attachment — Submitted filename: Comments for PONE-D-25-58376.docx [file pone.0348029.s001.docx]

Comments for: Spatial Distribution and Determinants of Unimproved Sanitation Facilities Among Households in Somalia: Using Somalia Integrated Household Budget Survey (SIHBS 2022)

In general, the manuscript has some good information. English writing is acceptable. The manuscript can be accepted for publication after having some revisions as followings:

- Abstract is too long. This should be shortened, in particular the background and method.
- Introduction: Please use some updated reference (within the past 10 years).
- Study area: Should provide brief information about population, population density, economic growth, etc.
- Section 2.2 and 2.3 should combine into one section to avoid repetition. Additionally, please provide brief information about the type of survey: interview or questionnaire forms, online or offline, etc.
- Section 3.1 needs to include:
  - Discussion on the result for each variable. For instance, why the improved sanitation facilities were high in urban, low in rural; why it is different for marital status, etc.
  - Comparison with previous studies in Somali or similar countries in Africa on the status of unimproved or improved sanitation.
- Section 3.2 needs to have a discussion on which Model is more reliable, why there were the difference/similarity among Model 1 and Model 3.
- Section 3.4.1, provide deeper discussion on why the improved sanitation facilities were high in Lower Shabelle or Banadir regions and low in Bay or Hiraan regions;
- Section 3.4.2 should compare the normal distribution in Somali with previous studies.
- The Discussion section: should state more about why there were such results. Some aspects as economic condition, level of public education, etc. should be analysed in details to support the results. After that, please propose some solutions to improve this issue.
- The conclusion should be rewritten with the information added from the discussion.

Conclusion: Major revision is required.
