# Supplementary material for: Spatial distribution and determinants of unimproved sanitation facilities among households in Somalia: Using Somalia integrated household budget survey (SIHBS 2022)
Source: PLoS One. 2026 Apr 24;21(4):e0348029. doi: 10.1371/journal.pone.0348029 (PMC13108892; doi:10.1371/journal.pone.0348029)
Supplement: Attachment — Submitted filename: Point by poin Response Letter.docx [file pone.0348029.s003.docx]

**Point-by-Point Response Letter**

Date: February 21, 2026

To: The Editorial Office,
PLOS ONE

**Manuscript ID:** PONE-D-25-58376
**Manuscript Title:** "Spatial Distribution and Determinants of Unimproved Sanitation Facilities Among Households in Somalia: Using Somalia Integrated Household Budget Survey (SIHBS 2022)"

Dear Dr. Alfredo Luis Fort,

Thank you for your email dated February 19, 2026, and for the opportunity to revise our manuscript. We are grateful to you and the reviewers for the insightful and constructive feedback. We believe the comments have allowed us to significantly improve the quality, clarity, and scientific rigor of our paper.

We have carefully addressed each point raised by the editor and both reviewers. The modifications in the revised manuscript are highlighted using the "Track Changes" feature. Below, we provide a detailed, point-by-point response to the comments.

We hope that the revised manuscript is now suitable for publication in PLOS ONE.

Sincerely,

Omar Muhumed Maidhane (On behalf of all authors)
Corresponding Author
Amoud University

**Response to Journal Requirements**

**1. PLOS ONE Style Requirements:**
**Comment:** Please ensure that your manuscript meets PLOS ONE's style requirements, including those for file naming.

**Response:** Acknowledged. We have thoroughly reviewed the PLOS ONE style templates and have revised the manuscript to ensure full compliance with all formatting and file naming conventions.

**2. Data Availability and Repository:**
**Comment:** The repository you have noted in your Data Availability statement does not qualify as an acceptable data repository. Please upload the minimal data set to a stable, public repository (such as figshare or Dryad).

**Response:** Thank you for this guidance. We have now deposited the minimal dataset necessary to replicate our study's findings in a PLOS-approved public repository (Figshare). The Data Availability Statement in the manuscript has been updated with the corresponding DOI to ensure full access.

**3. Copyrighted Map Images (Figures 1, 3, and 4):**
**Comment:** We cannot publish previously copyrighted maps. We require you to either present written permission from the copyright holder to publish these figures specifically under the CC BY 4.0 license, or remove/replace the figures.

**Response:** Thank you for your feedback. We confirm that Figures 1, 3, and 4 are original figures created by the authors using public domain data from Natural Earth and shapefiles from the Humanitarian Data Exchange (HDX).

**4. Figure 2 Quality:**
**Comment:** Please upload a new copy of Figure 2, as the detail is not clear.

**Response:** Acknowledged. We have generated a new, high-resolution version of Figure 2 that meets the journal's technical requirements for clarity and detail. This new figure has been uploaded with the revised submission.

**Response to Reviewer #1**

We sincerely thank Reviewer #1 for the positive assessment and valuable suggestions for improvement.

**1. Abstract Length:**
**Comment:** Abstract is too long. This should be shortened, in particular the background and method.

**Response:** Acknowledged. We have revised and condensed the abstract. The Background and Methods sections have been significantly shortened to be more concise, allowing the abstract to focus more sharply on the key findings and their implications, while adhering to the journal's word count guidelines.

**2. Updated References:**
**Comment:** Introduction: Please use some updated reference (within the past 10 years).

**Response:** Thank you for this important suggestion. We have reviewed the literature again and have updated the Introduction section to include more recent references, ensuring our study is framed within the most current scientific context.

**3. Study Area Description:**
**Comment:** Study area: Should provide brief information about population, population density, economic growth, etc.

**Response:** Acknowledged. We have expanded the "Study Area" section to include concise, relevant details about Somalia's population, population density, and recent economic context, providing readers with a better understanding of the study setting.

**4. Merging Sections 2.2 & 2.3 and Survey Type:**
**Comment:** Section 2.2 and 2.3 should combine into one section to avoid repetition. Additionally, please provide brief information about the type of survey: interview or questionnaire forms, online or offline, etc.

**Response:** We agree with this suggestion. Sections 2.2 and 2.3 have been merged into a single, streamlined section titled "Study Design and Data Source" to eliminate repetition. We have also added information clarifying that the data was collected via in-person household interviews using structured questionnaires.

**5. Discussion in Section 3.1 (Univariate Analysis):**
**Comment:** Section 3.1 needs to include discussion on the result for each variable... and comparison with previous studies...

**Response:** Acknowledged. We have expanded the first paragraph of the Results section (formerly Section 3.1) to include a brief discussion of the most significant descriptive findings, such as the disparities between urban and rural areas. Furthermore, we have integrated comparisons with findings from similar studies in Somalia and other sub-Saharan African countries into the main Discussion section to provide a richer context for our results.

**6. Discussion on Model Reliability in Section 3.2 (Multilevel Analysis):**
**Comment:** Section 3.2 needs to have a discussion on which Model is more reliable, why there were the difference/similarity among Model 1 and Model 3.

**Response:** Thank you for pointing this out. We have added a dedicated subsection ("Model Comparison") following the multilevel analysis results. This new section explicitly discusses the model fit statistics (AIC, BIC, ICC), explaining why the Full Model (Model 3) is the most reliable and how the inclusion of both individual and community-level factors provides a more comprehensive explanation of the variance in unimproved sanitation.

**7. Deeper Discussion on Spatial Distribution (Section 3.4.1):**
**Comment:** Provide deeper discussion on why the improved sanitation facilities were high in Lower Shabelle or Banadir regions and low in Bay or Hiraan regions.

**Response:** Acknowledged. In the main Discussion section, we have now included a more detailed analysis of the spatial findings. We hypothesize potential reasons for the observed hotspots (e.g., conflict, displacement, and governance challenges in Bay and Hiraan) and coldspots (e.g., greater urbanization, government presence, and humanitarian investment in Banadir and Lower Shabelle).

**8. Comparison of Moran's I (Section 3.4.2):**
**Comment:** Section 3.4.2 should compare the normal distribution in Somali with previous studies.

**Response:** We have revised the discussion of the spatial analysis. We now clarify that while our Global Moran's I indicated a random *global* pattern, the significant *local* clustering (identified by Local Moran's I and Getis-Ord Gi*) is a common finding in public health geography. We now discuss this finding in the context of other studies that show health and infrastructure disparities are often highly localized rather than following a broad, country-wide pattern.

**9. Expansion of the Discussion Section:**
**Comment:** The Discussion section: should state more about why there were such results... Some aspects as economic condition, level of public education, etc. should be analysed... After that, please propose some solutions...

**Response:** This is a crucial point. The Discussion section has been substantially rewritten and expanded. We now delve deeper into the socio-economic drivers behind our findings, analyzing the roles of poverty, education, displacement, and rapid urbanization. Crucially, we have added a new paragraph proposing specific, evidence-based policy recommendations and solutions tailored to address the challenges identified in our analysis.

**10. Rewriting the Conclusion:**
**Comment:** The conclusion should be rewritten with the information added from the discussion.

**Response:** Acknowledged. In line with the expanded Discussion, the Conclusion has been rewritten to be more impactful. It now better synthesizes the key findings and directly links them to the urgent policy implications and proposed solutions, providing a stronger concluding statement for the manuscript.

**Response to Reviewer #2**

We thank Reviewer #2 for the rigorous and detailed feedback, which has been instrumental in strengthening our manuscript's methodology and interpretation.

**1. Title Length:**
**Comment:** The title is fine, but I think it's a little long. I suggest this: Spatial distribution and multilevel determinants of unimproved sanitation in Somalia: evidence from SIHBS 2022.

**Response:** Thank you for the excellent suggestion. We agree that a more concise title is better. We have adopted a revised title very similar to your recommendation: "Spatial Distribution and Multilevel Determinants of Unimproved Sanitation in Somalia: Evidence from the 2022 Integrated Household Budget Survey."

**2. Inconsistency Between Descriptive and Multilevel Results:**
**Comment:** Inconsistency between the descriptive results and those of the multilevel model concerning the use of unimproved sanitation facilities (e.g., Nomadic: 83.28% prevalence vs. AOR 0.04; Urban: 23.88% prevalence vs. AOR 7.99).

**Response:** This is a critical point that requires clarification. We have added a detailed explanation in the Discussion section.

- **For Nomadic Populations:** The descriptive statistic (83.28%) reflects the high prevalence of unimproved sanitation, which is dominated by the practice of open defecation. However, the multilevel model's Adjusted Odds Ratio (AOR) of 0.04 is relative to the rural reference group and controls for other factors (like wealth and region). This AOR indicates that nomads are significantly *less likely to use a built unimproved facility* (e.g., a pit latrine without a slab) than rural dwellers, precisely because open defecation is the norm and they often lack any physical facility at all.
- **For Urban Populations:** The AOR of 7.99 indicates that, after controlling for individual-level factors like wealth and education, living in an urban area is associated with significantly *higher odds* of using an unimproved facility compared to living in a rural area. This highlights the crisis of unplanned urbanization, where infrastructure has not kept pace with population growth, leading to crowded settlements with poor sanitation even when residents might be better off in other respects. We have ensured this paradox is now clearly explained in the manuscript.

**3. Justification for Combined Spatial and Multilevel Analysis:**
**Comment:** Justify why spatial + multilevel analysis adds scientific value. Clarify what distinguishes this study from previous ones.

**Response:** Thank you for this prompt. We have revised the Introduction to more explicitly state the novelty and scientific value of our dual approach. We now emphasize that this study is among the first in Somalia to combine these methods, which allows us to: (1) Simultaneously assess the effects of individual, household, and community-level factors, and (2) Pinpoint the specific geographic locations where the burden of poor sanitation is highest. This provides a more robust and actionable evidence base for policymakers than either method would alone.

**4. Clarification on Open Defecation:**
**Comment:** Open defecation is classified as unimproved. However, you then explain that low nomadic AOR is due to unrecorded open defecation. This contradicts your own definition. To be clarified.

**Response:** We apologize for the lack of clarity. We have revised the text to be more precise. Our definition of "unimproved sanitation" in the Methods section correctly includes open defecation. The explanation in the Discussion regarding the low nomadic AOR has been rephrased. We now clarify that the model is distinguishing between different types of unimproved sanitation. The low AOR reflects that nomads are less likely to have a *constructed* unimproved facility compared to the reference group, as open defecation (having no facility) is their predominant practice. The wording has been corrected to avoid any contradiction.

**5. Methodological Rigor of Kriging:**
**Comment:** The section on kriging lacks methodological rigor... no cross-validation... variogram is not described...

**Response:** We acknowledge this significant methodological shortcoming in our original submission. We have substantially revised the "Spatial Analysis" subsection within the Methods. We have now included:

- A description of the empirical variogram model used for ordinary kriging.
- Details of the model's parameters (nugget, sill, and range).
- The results of a cross-validation analysis, including key performance indicators (e.g., Root Mean Square Error), to validate the predictive performance and assess the spatial uncertainty of our interpolation map. This adds the necessary rigor and reproducibility to our spatial analysis.

**6. Correction of "Result" vs. "Results":**
**Comment:** Correct inconsistencies between “Result” and “Results.”

**Response:** Acknowledged. We have carefully proofread the entire manuscript and have corrected this and other typographical errors to ensure consistency throughout the text.
